# Duration of Adulthood Overweight, Obesity, and Cancer Risk in the Women’s Health Initiative: A Longitudinal Study from the United States

**DOI:** 10.1371/journal.pmed.1002081

**Published:** 2016-08-16

**Authors:** Melina Arnold, Luohua Jiang, Marcia L. Stefanick, Karen C. Johnson, Dorothy S. Lane, Erin S. LeBlanc, Ross Prentice, Thomas E. Rohan, Beverly M. Snively, Mara Vitolins, Oleg Zaslavsky, Isabelle Soerjomataram, Hoda Anton-Culver

**Affiliations:** 1 Section of Cancer Surveillance, International Agency for Research on Cancer, Lyon, France; 2 Department of Epidemiology, School of Medicine, University of California, Irvine, California, United States of America; 3 Stanford Prevention Research Center, Department of Medicine, Stanford University, Stanford, California, United States of America; 4 Department of Preventive Medicine, University of Tennessee Health Science Center, Memphis, Tennessee, United States of America; 5 Department of Preventive Medicine, Stony Brook University School of Medicine, Stony Brook, New York, United States of America; 6 Center for Health Research, Kaiser Permanente, Portland, Oregon, United States of America; 7 Division of Public Health Sciences, Fred Hutchinson Cancer Research Center, Seattle, Washington, United States of America; 8 Department of Epidemiology and Population Health, Albert Einstein College of Medicine, Bronx, New York, United States of America; 9 Division of Public Health Sciences, Wake Forest School of Medicine, Winston-Salem, North Carolina, United States of America; 10 Faculty of Health Sciences and Social Welfare, University of Haifa, Haifa, Israel; London School of Hygiene & Tropical Medicine, UNITED KINGDOM

## Abstract

**Background:**

High body mass index (BMI) has become the leading risk factor of disease burden in high-income countries. While recent studies have suggested that the risk of cancer related to obesity is mediated by time, insights into the dose-response relationship and the cumulative impact of overweight and obesity during the life course on cancer risk remain scarce. To our knowledge, this study is the first to assess the impact of adulthood overweight and obesity duration on the risk of cancer in a large cohort of postmenopausal women.

**Methods and Findings:**

Participants from the observational study of the Women’s Health Initiative (WHI) with BMI information from at least three occasions during follow-up, free of cancer at baseline, and with complete covariate information were included (*n =* 73,913). Trajectories of BMI across ages were estimated using a quadratic growth model; overweight duration (BMI ≥ 25 kg/m^2^), obesity duration (BMI ≥ 30 kg/m^2^), and weighted cumulative overweight and obese years, which take into account the degree of overweight and obesity over time (a measure similar to pack-years of cigarette smoking), were calculated using predicted BMIs. Cox proportional hazard models were applied to determine the cancer risk associated with overweight and obesity duration. In secondary analyses, the influence of important effect modifiers and confounders, such as smoking status, postmenopausal hormone use, and ethnicity, was assessed. A longer duration of overweight was significantly associated with the incidence of all obesity-related cancers (hazard ratio [HR] per 10-y increment: 1.07, 95% CI 1.06–1.09). For postmenopausal breast and endometrial cancer, every 10-y increase in adulthood overweight duration was associated with a 5% and 17% increase in risk, respectively. On adjusting for intensity of overweight, these figures rose to 8% and 37%, respectively. Risks of postmenopausal breast and endometrial cancer related to overweight duration were much more pronounced in women who never used postmenopausal hormones. This study has limitations because some of the anthropometric information was obtained from retrospective self-reports. Furthermore, data from longitudinal studies with long-term follow-up and repeated anthropometric measures are typically subject to missing data at various time points, which was also the case in this study. Yet, this limitation was partially overcome by using growth curve models, which enabled us to impute data at missing time points for each participant.

**Conclusions:**

In summary, this study showed that a longer duration of overweight and obesity is associated with an increased risk of developing several forms of cancer. Furthermore, the degree of overweight experienced during adulthood seemed to play an important role in the risk of developing cancer, especially for endometrial cancer. Although the observational nature of our study precludes inferring causality or making clinical recommendations, our findings suggest that reducing overweight duration in adulthood could reduce cancer risk and that obesity prevention is important from early onset. If this is true, health care teams should recognize the potential of obesity management in cancer prevention and that excess body weight in women is important to manage regardless of the age of the patient.

## Abstract 2

In a longitudinal study, Melina Arnold and colleagues assess the relationship between adulthood overweight and obesity duration and cancer risks in postmenopausal women.

## Author Summary

**Why Was This Study Done?:**

- Excess weight has become the leading risk factor for disease burden in high-income countries and has been offsetting or surpassing the decreasing disease burden attributable to tobacco smoking. Excess weight has been linked to the development of several types of cancer.
- To date, most studies exploring the relationship between excess weight and cancer risk looked at cross-sectional exposure information on overweight and obesity, i.e., height and weight measured at one point in time. Insights into the dose-response relationship of the cumulative impact of overweight and obesity during the life course on cancer risk remain scarce.

**What Did the Researchers Do and Find?:**

- This study examined how the timing, duration, and intensity of overweight and obesity during adulthood impacts on cancer risk, taking into account important information on other factors related to obesity, such as physical activity, diet, smoking, hormone use, and diabetes history.
- A total of 73,913 women were included in the study, and 6,301 obesity-related cancers were diagnosed during a mean follow-up of 12.6 y. About two-thirds of all included women were ever overweight or obese during adulthood.
- The study found that being overweight for a longer duration during adulthood significantly increased the incidence of all obesity-related cancers by 7% (for every ten-year increase in adulthood overweight duration), of postmenopausal breast cancer by 5%, and of endometrial cancer by 17%. After adjusting for the intensity of overweight (that is, how overweight individuals were), these figures rose to 8% for postmenopausal breast cancer and 37% for endometrial cancer (for every ten years spent with body mass index ten units above normal weight).

**What Do These Findings Mean?:**

- How much of their adult lives women are overweight and how overweight they are play important roles in cancer risk. This finding highlights the importance of obesity prevention at all ages and from early onset.

## Introduction

High body mass index (BMI) has become the leading risk factor for disease burden in high-income countries and has been offsetting or surpassing the decreasing disease burden attributable to tobacco smoking [1]. In the US, currently about 70% of all adults are considered overweight (BMI ≥ 25 kg/m^2^) and 36% obese (BMI ≥ 30 kg/m^2^, according to WHO classification), making the US one of the countries with the highest prevalence of obesity [2]. This development has also been found to contribute substantially to the country’s poor international ranking in longevity [3,4]. Continuing increases in obesity prevalence seen over the past decades remain of great public health concern.

Overweight and obesity have been associated with an increased risk of and mortality from cancer and other chronic diseases such as type 2 diabetes and cardiovascular disease [5]. In the US, more than 100,000 cancer cases were attributed to high BMI in the year 2012 alone [6]. Cancers previously linked to high BMI include postmenopausal breast cancer, adenocarcinoma of the esophagus, and pancreatic, colorectal, renal, endometrial, ovarian, and gallbladder cancer [7,8]. To date, most studies of these associations have used cross-sectional exposure information on BMI, i.e., BMI measured at one point in time and typically obtained at recruitment. Yet, insights into the dose-response relationship of the cumulative impact of overweight and obesity during the life course on cancer risk remain scarce. While age-dependent and cumulative effects of weight change have previously been reported to affect the risk of postmenopausal breast cancer [9–11], only a few studies have investigated the association between overweight duration and cancer outcomes [9,12]. It is thus still unclear how different exposure durations of overweight and obesity are associated with cancer development.

In this study, we assessed the impact of adulthood overweight and obesity duration and cumulative intensity on cancer risk in US women, using data from the observational study cohort of the Women’s Health Initiative (WHI). In secondary analyses, we investigated the influence of important effect modifiers and confounders, such as smoking status, postmenopausal hormone use, and ethnicity.

## Methods

### Ethics

WHI procedures and protocols were approved by the institutional review boards at each participating institution, and all participants provided written informed consent.

### Study Population

**Fig 1:**
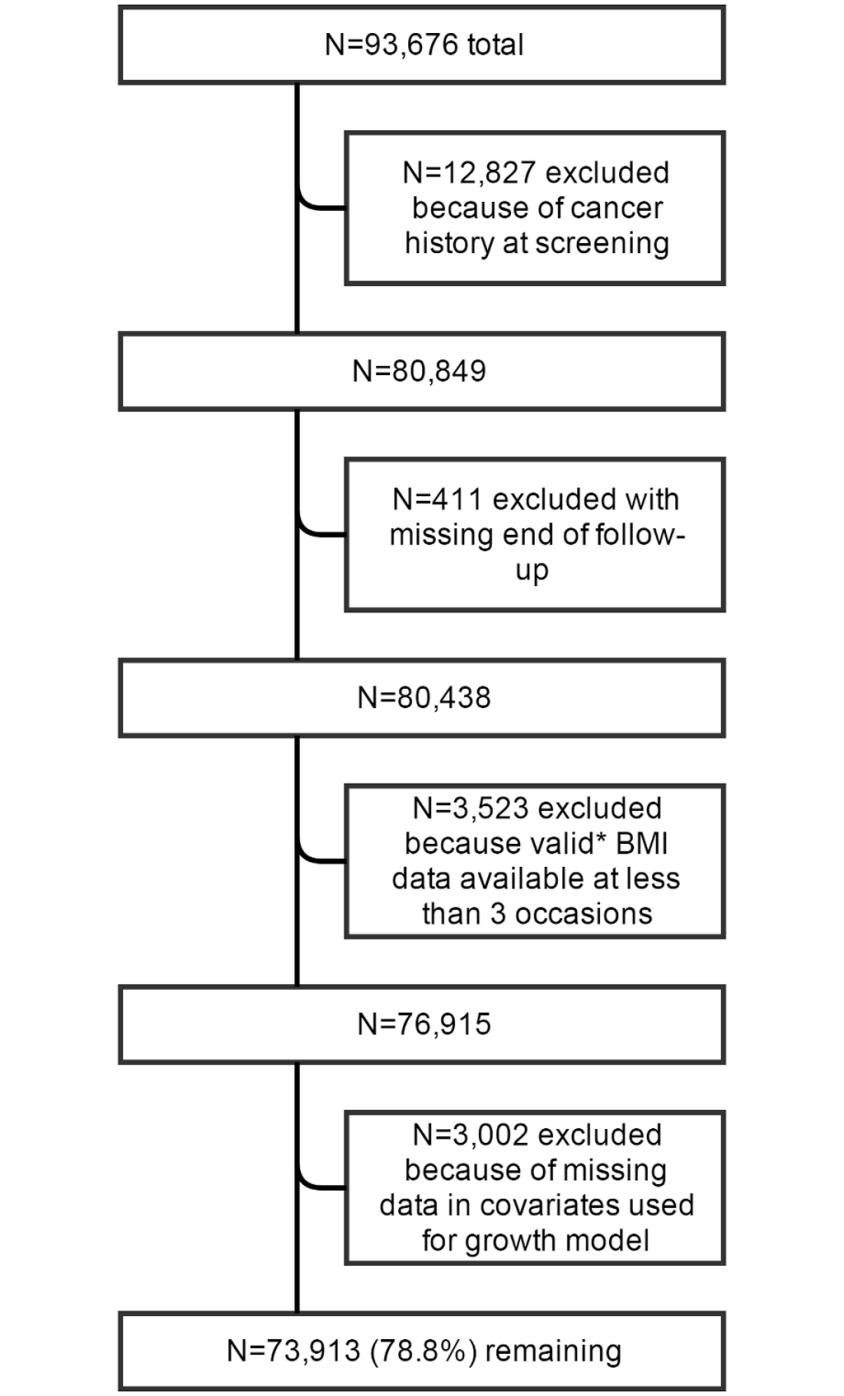
Flowchart of participant inclusion. *Including data at baseline and excluding data from the year preceding cancer diagnosis; including BMI from self-reported as well as measured height and weight; BMI values lower <15 or >70 kg/m^2^ were excluded.

The WHI is a large, multi-center prospective cohort study of postmenopausal women. The WHI was designed to have a clinical trial arm and an observational study cohort [13], and recruited postmenopausal women aged 50–79 y at 40 clinical centers nationwide between October 1, 1993, and December 21, 1998, to be followed for the development of diseases that are the most common causes of death, including cardiovascular disease and cancer. Details of the study design and methods have been published elsewhere [13–16]. In total, 93,676 women were enrolled in the observational study, and 68,132 were enrolled in the clinical trial arm (*n =* 161,808) [16]. For this study, we included all participants from the observational study cohort, except those who reported cancer prior to or at baseline or without data on cancer history (*n =* 12,827) and women with incomplete follow-up information (*n =* 411) (Fig 1).

### Anthropometric Assessment

Information on BMI for was obtained from retrospective self-reports at baseline for ages 18, 35, and 50 y, from weight and height measurements at baseline and at 3-y follow-up, and from self-reports at follow-up years 4–8. BMI was calculated by dividing weight in kilograms by height in meters squared. For inclusion in the study, women were required to have valid body weight information from at least three occasions and a valid baseline measurement of body weight and height.

### Covariate Assessment

Covariates included baseline information on age (continuous), ethnicity (six categories), education (11 categories), energy intake (in kilocalories, continuous), diet quality (Alternate Mediterranean Diet Score [17], nine categories), physical activity (frequency of moderate exercise per week, four categories), smoking status (never/past/current smoker), and outcome-specific covariates—for pancreatic cancer: diabetes (never/ever diabetic); for colon cancer: diabetes and red meat intake (servings/day); for postmenopausal breast, ovarian, and endometrial cancer: postmenopausal hormone use (never/past/current user), number of full-term pregnancies (continuous), age at first term pregnancy (<20, 20–24, 25–29, 30–34, 35–39, 40–44, 45+ y), and age at menopause (continuous).

### Case Ascertainment

Adjudication and outcome ascertainment for the WHI have been described elsewhere [18]. In brief, all outcomes were self-reported annually in the observational study arm. Only first invasive cancers confirmed by adjudication were included in these analyses. All analyses were conducted for the following cancers with convincing evidence of a positive relation with excess BMI [7,8]: colon, rectum, liver, gallbladder, pancreas, postmenopausal breast, endometrium, ovary, kidney, and thyroid, as well as the combination of all of these, termed “all obesity-related cancers.”

### Statistical Analysis

The analysis was carried out in two steps. In the first step, BMI was modeled across all ages using a quadratic growth model with random intercept and random slope, incorporating all available BMI information from all included participants [19]. No random coefficient was included for the quadratic term.

BMI information in the year preceding cancer diagnosis for those who developed an invasive malignancy was excluded. This model was developed and adjusted in a stepwise manner by adding ethnicity, education, and baseline physical activity, smoking status, energy intake, and diet quality score to the model. Using this approach, we allowed individuals to have their own BMI trajectory. Using the full model, BMI was predicted from age 18 y until the age at study exit for every cohort member. The obtained predicted age-specific BMI data were then used to compute the following parameters: overweight (BMI ≥ 25 kg/m^2^) and obesity (BMI ≥ 30 kg/m^2^) duration in years and weighted cumulative overweight years (OWY) and obese years (OBY). OWY and OBY were calculated by multiplying the duration of overweight and obesity in years by the difference (in BMI units) above normal BMI (≥25 kg/m^2^) for overweight and above overweight (≥30 kg/m^2^) for obesity for each age. This allowed us to take the degree of each participant’s overweight and obesity over time into account. An individual with a BMI of 35 kg/m^2^ for 10 y would thus contribute 100 (= 10 × [35 − 25]) OWY and 50 (= 10 × [35 − 30]) OBY units. The merit of this approach as a better predictor than cross-sectional BMI information alone for many obesity-related outcomes has been described earlier [20]. Overweight and obesity duration were assessed per 10-y increments and OWY and OBY per 100 units.

In the second step of the analysis, Cox proportional hazard models with time since enrollment as the underlying time metric were fitted to estimate hazard ratios (HRs) and 95% confidence intervals (CIs) for the relationship between BMI overweight/obesity duration, OWY/OBY, and the risk of developing specific cancers. Overweight/obesity duration and OWY/OBY were treated as continuous, time-varying covariates. Participants were censored at study exit due to death, loss to follow-up, any cancer diagnosis, or end of follow-up (August 29, 2014), whichever occurred first. Three models were fitted for all outcomes with adjustments for several established risk factors for obesity-related cancers. In model 1, adjustment was made for age. Model 2 was additionally adjusted for ethnicity and education. In model 3, smoking status, physical activity, energy intake, and diet quality score were additionally introduced into the model. Cancer-specific adjustments were made in model 4, introducing age at first birth, age at menopause, parity, and postmenopausal hormone use for breast, endometrial, and ovarian cancer; red meat intake and diabetes status for colon cancer; and diabetes status for pancreatic cancer. Possible interactions were tested by fitting models with and without the interaction term, and corresponding *p*-values were computed from likelihood ratio tests comparing the two models. In order to assess the potentially nonlinear dose-response relationships between overweight duration, OWY, and cancer risk, we used restricted cubic splines with three knots to model those relationships [21]. Nonlinearity was evaluated by testing the null hypothesis that the coefficients of the splines were the same.

In secondary analyses, we assessed a priori interactions by stratifying by postmenopausal hormone use, hysterectomy and/or oophorectomy, ethnicity, diabetes, and smoking status. We also tested the robustness of our findings by predicting BMI trajectories using self-reported height and weight assessments only.

All analyses were carried out using Stata 13.

## Results

The final sample for analyses included 73,913 women with a mean follow-up of 12.6 y (standard deviation [SD] = 5.1), during which a total of 6,301 invasive obesity-related cancer cases occurred. Out of all included study participants, 40% (*n =* 29,770) were never overweight (BMI < 25 kg/m^2^) during their adult life (age 18 y until study exit), and 60% (*n =* 44,143) were ever overweight (BMI ≥ 25 kg/m^2^), almost half of whom (*n =* 19,654) were also ever obese (BMI ≥ 30 kg/m^2^) (Table 1). These values were estimated based on BMI data from on average >9 occasions (both measured and self-reported) per study participant. Women who were ever overweight were on average overweight for 31.3 y; those who were ever obese were, on average, obese for 20.6 y. Compared with women who were never overweight, those who were ever overweight or obese were slightly younger at baseline, had a lower education, and were more likely to be African-American (Table 1). While no differences existed in smoking status, women who were ever overweight or obese were less physically active, consumed more calories, had a lower diet quality score, and more often reported ever being diabetic than women who were never overweight. Women who were never overweight were more likely to be using postmenopausal hormones and less often had a hysterectomy.

**Table 1:** Cohort characteristics at recruitment by adulthood overweight and obesity.

Characteristic	All Participants, *n =* 73,913		Never Overweight* (BMI < 25 kg/m^2^), *n =* 29,770		Ever Overweight* (BMI ≥ 25 kg/m^2^), *n =* 44,143		Ever Obese* (BMI ≥ 30 kg/m^2^), *n =* 19,654	
	Mean or *n*	SD or Percent	Mean or *n*	SD or Percent	Mean or *n*	SD or Percent	Mean or *n*	SD or Percent
**Follow-up (y)**	12.6	5.1	12.7	5.1	12.6	5.2	12.6	5.1
**Age (y)**	63.3	7.3	64.2	7.4	62.7	7.2	61.8	6.9
**BMI (kg/m** ^**2**^ **)**	27.2	5.8	22.6	2.2	30.3	5.5	34.2	5.5
**Total number of anthropometric assessments**	9.4	2.0	9.6	1.9	9.3	2.0	9.3	2.1
Number measured	1.9	0.5	1.9	0.5	1.9	0.5	1.9	0.5
Number self-reported	7.6	1.7	7.7	1.7	7.5	1.8	7.4	1.8
**Race/ethnicity**								
American Indian or Alaska Native	307	0.4%	81	0.3%	226	0.5%	128	0.7%
Asian or Pacific Islander	2,276	3.1%	1,424	4.8%	852	1.9%	226	1.1%
African-American	5,811	7.9%	951	3.2%	4,860	11.0%	2,958	15.1%
Hispanic/Latino	2,592	3.5%	716	2.4%	1,876	4.2%	902	4.6%
White (not of Hispanic origin)	62,097	84.0%	26,297	88.3%	35,800	81.1%	15,205	77.4%
Other	830	1.1%	301	1.0%	529	1.2%	235	1.2%
**Education**								
High school diploma/GED or less	3,472	4.7%	903	3.0%	2,569	5.8%	1,425	7.3%
School after high school	39,074	52.9%	14,237	47.8%	24,837	56.3%	11,512	58.6%
College degree or higher	31,367	42.4%	14,630	49.1%	16,737	37.9%	6,717	34.2%
**Smoking**								
Never smoker	37,904	51.3%	15,579	52.3%	22,325	50.6%	9,858	50.2%
Past smoker	31,455	42.6%	12,236	41.1%	19,219	43.5%	8,666	44.1%
Current smoker	4,554	6.2%	1,955	6.6%	2,599	5.9%	1,130	5.7%
**Physical activity**								
No activity	9,925	13.4%	2,597	8.7%	7,328	16.6%	4,177	21.3%
Some activity of limited duration	28,250	38.2%	10,170	34.2%	18,080	41.0%	8,501	43.3%
2 to <4 episodes per week^†^	13,598	18.4%	5,611	18.8%	7,987	18.1%	3,334	17.0%
4 episodes per week^†^	22,140	30.0%	11,392	38.3%	10,748	24.3%	3,642	18.5%
**Energy intake (kcal)**	1,548.4	685.5	1,468.5	570.9	1,602.5	748.4	1,683.9	848.5
**Diet quality score** ^‡^								
<4	28,603	38.7%	9,837	33.0%	18,766	42.5%	9,060	46.1%
4–6	37,709	51.0%	15,908	53.4%	21,801	49.4%	9,307	47.4%
7–9	7,601	10.3%	4,025	13.5%	3,576	8.1%	1,287	6.5%
**Diabetes**								
Never	69,932	94.6%	29,062	97.6%	40,870	92.6%	17,519	89.1%
Ever	3,920	5.3%	680	2.3%	3,240	7.3%	2,120	10.8%
Missing	61	0.1%	28	0.1%	33	0.1%	15	0.1%
**Postmenopausal hormone use**								
None	21,091	28.5%	7,485	25.1%	13,606	30.8%	6,698	34.1%
Past user	14,248	19.3%	5,424	18.2%	8,824	20.0%	4,125	21.0%
Current user	37,389	50.6%	16,385	55.0%	21,004	47.6%	8,536	43.4%
Missing	1,185	1.6%	476	1.6%	709	1.6%	295	1.5%
**Hysterectomy**								
Never	44,476	60.2%	19,093	64.1%	25,383	57.5%	10,858	55.2%
Ever	29,379	39.7%	10,649	35.8%	18,730	42.4%	8,783	44.7%
Missing	58	0.1%	28	0.1%	30	0.1%	13	0.1%
**Oophorectomy (bilateral)**								
Never	58,726	79.5%	24,155	81.1%	34,571	78.3%	15,110	76.9%
Ever	13,838	18.7%	5,162	17.3%	8,676	19.7%	4,087	20.8%
Missing	1,349	1.8%	453	1.5%	896	2.0%	457	2.3%
**Number of term pregnancies**								
Never been pregnant/no term pregnancies	9,196	12.4%	4,126	13.9%	5,070	11.5%	2,305	11.7%
1	6,586	8.9%	2,687	9.0%	3,899	8.8%	1,753	8.9%
2	19,457	26.3%	8,406	28.2%	11,051	25.0%	4,676	23.8%
3	17,814	24.1%	7,239	24.3%	10,575	24.0%	4,483	22.8%
4 or more	20,486	27.7%	7,153	24.0%	13,333	30.2%	6,337	32.2%
Missing	374	0.5%	159	0.5%	215	0.5%	100	0.5%
**Age at first term pregnancy** ^§^								
<30 y	52,378	90.3%	20,637	89.0%	31,741	91.2%	14,131	91.9%
≥30 y	5,604	9.7%	2,557	11.0%	3,047	8.8%	1,247	8.1%
**Age at menopause**	48.3	6.3	48.7	6.0	48.0	6.5	47.7	6.7

*Derived from growth model and based on predicted BMI values from age 18 y until the end of follow-up. By definition, the group of ever overweight (BMI ≥ 25 kg/m^2^) includes the ever obese (BMI ≥ 30 kg/m^2^).

^†^Episodes per week of moderate and strenuous recreational physical activity of ≥20 min duration (includes walking fairly fast or very fast, moderate physical activity, and strenuous physical activity).

^‡^Alternate Mediterranean Diet Score, based on nine food groups.

^§^In parous women only.

GED, general educational development.

A longer overweight duration significantly increased the risk of all obesity-related cancers combined (multivariable-adjusted HR per 10-y increment: 1.07, 95% CI 1.06–1.09) (Table 2). The strongest associations were observed for endometrial (HR 1.17, 95% CI 1.12–1.22) and kidney cancer (HR 1.16, 95% CI 1.07–1.26), while no significant associations were found for rectal, liver, gallbladder, pancreatic, ovarian, and thyroid cancer. When taking into account the degree of overweight over time, the association became stronger for all obesity-related cancers combined, with an increase in risk of 12% for every 100 units of OWY. For endometrial cancer, the HR per 100 OWY was 1.37 (95% CI 1.29–1.46). The results for obesity duration were even more pronounced and showed a significant association for all obesity-related cancers combined (multivariable-adjusted HR per 10-y increment: 1.10, 95% CI 1.08–1.12) and individually for colon, breast, endometrial, and kidney cancer, with HRs for every 10 y of obesity ranging from 1.07 (95% CI 1.04–1.10) for breast cancer to 1.23 (95% CI 1.18–1.28) for endometrial cancer. Risks associated with OBY were similar. Further cancer-specific adjustments (for pancreatic cancer: diabetes; for colon cancer: diabetes and red meat intake; for postmenopausal breast, ovarian, and endometrial cancer: postmenopausal hormone use, number of full-term pregnancies, age at first term pregnancy, and age at menopause) did not materially change these results (Table A in S1 Appendix).

**Table 2:** Hazard ratios of specific cancers related to overweight (BMI ≥ 25 kg/m^2^) and obesity (BMI ≥ 30 kg/m^2^) duration and intensity.

Cancer	Number of Cases	Overweight Duration (per 10 y)		OWY (per 100 Units)		Obesity Duration (per 10 y)		OBY (per 100 Units)	
		Age-Adjusted HR (95% CI)	MV-Adjusted HR (95% CI)*	Age-Adjusted HR (95% CI)	MV-Adjusted HR (95% CI)*	Age-Adjusted HR (95% CI)	MV-Adjusted HR (95% CI)*	Age-Adjusted HR (95% CI)	MV-Adjusted HR (95% CI)*
All obesity-related cancers^†^	6,301	1.06 (1.05–1.08)	1.07 (1.06–1.09)	1.11 (1.08–1.13)	1.12 (1.09–1.15)	1.09 (1.07–1.11)	1.10 (1.08–1.12)	1.11 (1.08–1.14)	1.12 (1.08–1.15)
All obesity-related cancers excluding breast^†^	2,578	1.09 (1.06–1.11)	1.09 (1.07–1.12)	1.17 (1.13–1.21)	1.17 (1.13–1.22)	1.13 (1.10–1.16)	1.13 (1.10–1.16)	1.17 (1.12–1.21)	1.17 (1.12–1.21)
Colon	770	1.12 (1.08–1.16)	1.12 (1.08–1.17)	1.17 (1.10–1.24)	1.16 (1.09–1.24)	1.12 (1.07–1.18)	1.12 (1.06–1.17)	1.11 (1.02–1.21)	1.09 (1.00–1.20)
Rectum	115	1.08 (0.97–1.20)	1.09 (0.97–1.21)	1.05 (0.87–1.27)	1.05 (0.86–1.28)	1.08 (0.94–1.24)	1.08 (0.94–1.25)	1.09 (0.86–1.39)	1.09 (0.85–1.40)
Liver	69	1.00 (0.87–1.14)	0.96 (0.83–1.11)	1.12 (0.90–1.39)	1.06 (0.84–1.33)	1.11 (0.95–1.31)	1.07 (0.89–1.27)	1.21 (0.99–1.48)	1.17 (0.93–1.46)
Gallbladder	25	1.01 (0.81–1.26)	0.95 (0.75–1.20)	1.25 (0.92–1.71)	1.18 (0.85–1.64)	1.12 (0.87–1.45)	1.06 (0.79–1.43)	1.15 (0.77–1.73)	1.09 (0.68–1.75)
Pancreas	276	1.04 (0.97–1.11)	1.05 (0.98–1.12)	1.02 (0.91–1.15)	1.04 (0.91–1.17)	1.04 (0.94–1.14)	1.05 (0.95–1.16)	1.05 (0.89–1.25)	1.07 (0.90–1.26)
Postmenopausal breast	3,723	1.04 (1.02–1.06)	1.05 (1.03–1.07)	1.06 (1.03–1.10)	1.08 (1.05–1.12)	1.05 (1.03–1.08)	1.07 (1.04–1.10)	1.06 (1.01–1.11)	1.07 (1.02–1.12)
Endometrium	632	1.14 (1.09–1.19)	1.17 (1.12–1.22)	1.33 (1.25–1.41)	1.37 (1.29–1.46)	1.22 (1.17–1.26)	1.23 (1.18–1.28)	1.28 (1.21–1.35)	1.29 (1.22–1.36)
Ovary	360	0.98 (0.92–1.04)	0.99 (0.93–1.06)	0.99 (0.89–1.11)	1.02 (0.91–1.14)	1.02 (0.93–1.12)	1.05 (0.95–1.14)	1.09 (0.95–1.25)	1.12 (0.98–1.28)
Kidney	184	1.18 (1.09–1.28)	1.16 (1.07–1.26)	1.20 (1.07–1.36)	1.17 (1.02–1.33)	1.16 (1.07–1.27)	1.14 (1.04–1.25)	1.16 (1.00–1.35)	1.13 (0.96–1.33)
Thyroid	147	1.00 (0.91–1.10)	1.02 (0.92–1.13)	1.05 (0.89–1.23)	1.08 (0.91–1.28)	1.02 (0.88–1.17)	1.04 (0.90–1.20)	1.00 (0.77–1.30)	1.02 (0.79–1.33)

*All obesity-related cancers comprises postmenopausal breast cancer as well as cancer of the colon, rectum, liver, gallbladder, pancreas, endometrium, ovary, kidney, and thyroid.

^†^Multivariable HR is adjusted for age, ethnicity, education, physical activity, smoking status, dietary intake (in kilocalories), and diet quality score.

MV, multivariable.

**Fig 2:**
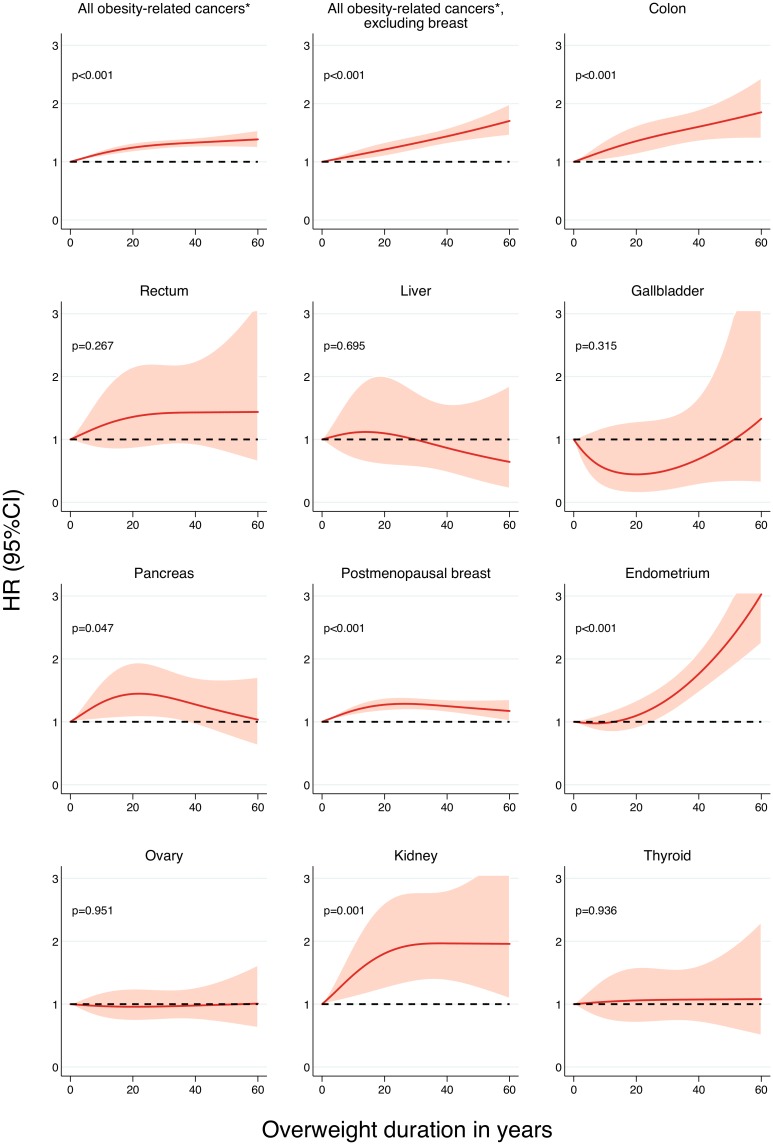
Association between overweight (BMI ≥ 25 kg/m^2^) duration since age 18 y and risk of specific cancers, allowing for non-linear effects, with 95% CIs. HRs are adjusted for age, ethnicity, education, physical activity, smoking status, dietary intake (in kilocalories), and diet quality score. Restricted cubic splines were fitted with knots at 0, 8, and 40 y. *p*-Values are for nonlinearity. *All obesity-related cancers comprises postmenopausal breast cancer as well as cancer of the colon, rectum, liver, gallbladder, pancreas, endometrium, ovary, kidney, and thyroid.

A dose-response relationship with increasing overweight duration was found for almost all cancers (Fig 2). The risk of endometrial cancer associated with increasing overweight duration rose in an exponential fashion, but became statistically significant only after 26 y of being overweight. This threshold effect disappeared when the degree of overweight over time was taken into account (Fig A in S1 Appendix). In contrast, the association of overweight duration with colon and postmenopausal breast cancer was linear.

**Fig 3:**
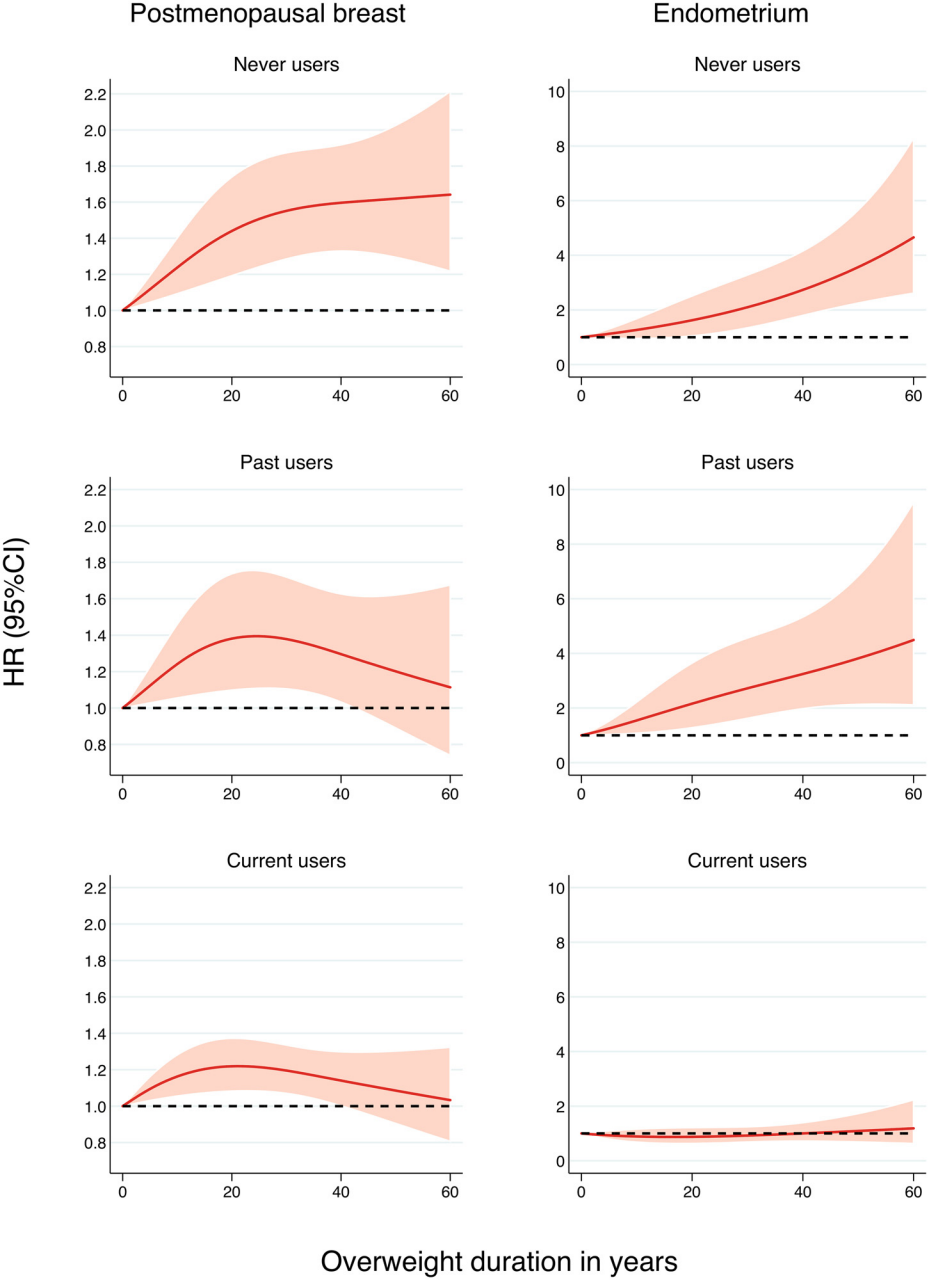
Association between overweight (BMI ≥ 25 kg/m^2^) duration since age 18 y and the risk of postmenopausal breast and endometrial cancer by postmenopausal hormone use, allowing for nonlinear effects, with 95% CIs. HRs are adjusted for age, ethnicity, education, physical activity, smoking status, dietary intake (in kilocalories), diet quality score, age at menopause, age at first birth, and parity. Restricted cubic splines were fitted with knots at 0, 15, and 44 y in never users; 0, 12, and 42 y in past users; and 0, 3, and 37 y in current users.

When stratifying the analyses by postmenopausal hormone use, for which statistically significant interactions were found, stronger associations of breast and endometrial cancer with all measures of overweight and obesity duration emerged in women who were never or past users (Table B in S1 Appendix). While the risk of endometrial cancer was statistically nonsignificant in current users, the risk of breast cancer was still elevated in this group, although at a much lower level than in never users (Fig 3). In line with these findings, the incidence rate of postmenopausal breast cancer was high in women who were ever overweight, regardless of their hormone use (Fig B in S1 Appendix). In contrast, among women who were never overweight in adulthood, current hormone users had a higher incidence rate of postmenopausal breast cancer than never or past postmenopausal hormone users. This pattern was somewhat different for endometrial cancer, where the incidence rate in ever overweight current users was similar to that in women who were never overweight and never or past hormone users.

Similar risks of postmenopausal breast, endometrial, and ovarian cancer associated with increasing overweight and obesity duration and intensity were found in women who had ever had a hysterectomy or oophorectomy compared to women who never underwent either of these procedures (Table C in S1 Appendix). Cancer risk related to increasing overweight and obesity duration was slightly higher in non-Hispanic white women than in African-American women for breast cancer (Table D in S1 Appendix), more pronounced in women who ever reported diabetes for colon cancer (Table E in S1 Appendix), and highest in current smokers compared to past and never smokers for colon and pancreatic cancer (Table F in S1 Appendix). Additional analyses using only self-reported BMI assessments confirmed the main findings (Table G in S1 Appendix).

## Discussion

Many previous studies have reported on the association between obesity and cancer, but, to our knowledge, this study is the first to assess the impact of adulthood overweight and obesity duration on the risk of various types of cancer in a large cohort of postmenopausal women. We found that longer durations of overweight and obesity were significantly associated with an increased incidence of obesity-related cancers, postmenopausal breast cancer, and colon, endometrial, and kidney cancer. Taking into account the intensity of overweight over time further increased the risks, and clear dose-response relationships were found. These findings are in line with previous studies on other chronic diseases, which showed that obesity duration is an important and independent predictor of type 2 diabetes [11], cardiovascular disease [10], and all-cause mortality [9]. Underlying biological mechanisms explaining these associations include a higher risk of developing hypertension and insulin resistance [10,11]. Earlier and long-term exposure to overweight and obesity may also increase the risk and severity of chronic inflammation, oxidative DNA damage, and alterations in endogenous hormone metabolism, three key mechanisms that have been found to be associated with increased risk of cancer [22].

We also observed that the risks of postmenopausal breast and endometrial cancer related to overweight and obesity duration were modified by postmenopausal hormone use and were largely attenuated or even eliminated among postmenopausal hormone users. Very high levels of estrogen in women using postmenopausal hormones have been postulated to obscure the effect of obesity and might explain this finding. While similar effect modification was reported in earlier analyses of the WHI observational study [23,24], in the Million Women Study [25–27], and in several other prospective cohorts [28–30], a recent analysis of the WHI clinical trial suggested a remaining positive association between obesity and postmenopausal cancer risk, independent of postmenopausal hormone use [31]. This finding was attributed to the often self-reported height, weight, and hormone use information in observational studies as well as outcome ascertainment bias. Strong associations found for endometrial cancer and its exponential dose-response relationship with overweight duration and intensity confirm risk patterns observed in previous studies [32,33].

Long-term follow-up and many repeated measurements of weight and height are necessary in order to fully capture lifetime overweight and obesity exposure and to quantify corresponding health effects. In our study, we used data from a large cohort of women with numerous repeated measurements and self-reports of weight and height for each individual, and applied a novel approach to estimate adulthood overweight and obesity duration. By modeling individual BMI trajectories across age using growth curves, all observed BMI information for an individual could be used, and unbalanced repeated measurements accommodated. Compared to traditional approaches to handling incomplete time-dependent data, such as linear interpolation and last observation carried forward methods, which may not adequately model BMI trajectories, growth models can incorporate any covariate data that might be important when predicting an individual’s BMI during life course. Using this approach, we were able to quantify the risk associated with both the duration and the intensity of overweight and obesity, as opposed to most previous studies, which focused on cross-sectional BMI information only.

Our study has limitations because of variation in how BMI information was collected and used. Some of this information was obtained from retrospective self-reports, which might be subject to measurement error. Yet, including only self-reported height and weight information when modeling BMI trajectories only marginally altered the results. Data from longitudinal studies with long-term follow-up and repeated anthropometric measures are typically subject to missing data at various time points due to periodical measurements, which was also the case in this study. Using growth curve models, we were able to impute data at missing time points for each participant. In a previous small-scale simulation study focusing on the effects of measurement errors, it was reported that using predicted biomarker values from growth curve models as a time-dependent covariate in Cox regression models—instead of applying a more naïve approach based on periodically observed biomarker values—yielded much less biased estimates for the association between the biomarker and the clinical outcome, especially when the variance in measurement errors was large [34].

BMI is not an ideal measure of body fatness as it does not differentiate tissue type (fatty, lean, bone). It has been suggested that other anthropometric measures such as waist circumference or waist-to-hip ratio better predict obesity-related health outcomes than BMI does [35,36]. These measures were, however, not available in a repeated fashion in the WHI. Additionally, BMI has limitations with regard to its application across different race/ethnic groups and ages [37]. In the US, African-American and Hispanic women have been reported to be more likely to be obese than non-Hispanic white and Asian women, while Asian women have been shown to have a higher percentage of body fat than non-Hispanic white women at similar BMI levels [38]. It might thus be appropriate to use different BMI thresholds for overweight and obesity in different race/ethnic groups. In our study, the risks for postmenopausal breast cancer associated with a longer overweight duration were higher in non-Hispanic white women than in African-American women. The fairly small number of women with an ethnicity other than non-Hispanic white did not allow further investigation of this issue. Furthermore, it is important to note that BMI serves as a proxy for many internal physiological processes that are the actual correlates of cancer development and that these also depend on many other lifestyle and environmental factors. Any residual confounding can therefore not be ruled out.

In summary, this study showed that the risk of cancer associated with overweight and obesity compounds over time, and a longer duration of overweight and obesity during adulthood is associated with increased risks of several cancers. Furthermore, not only the duration but also the degree of overweight seems to play an important role in the risk of developing cancer, especially for endometrial cancer. Although the observational nature of our study precludes inferring causality or making clinical recommendations, our findings suggest that reducing overweight duration in adulthood could reduce cancer risk. If this is true, health care teams should recognize the potential of obesity management in cancer prevention and that excess body weight in women is important to manage regardless of the age of the patient.

## Supporting Information

S1 Appendix Supplementary tables and figures. (DOCX)

S1 Analysis Plan (DOCX)

S1 STROBE Checklist (DOCX)

## Abbreviations

BMI: body mass index
CI: confidence interval
HR: hazard ratio
OBY: weighted cumulative obese years
OWY: weighted cumulative overweight years
SD: standard deviation
WHI: Women’s Health Initiative
