# Supplementary material for: Duration of Adulthood Overweight, Obesity, and Cancer Risk in the Women’s Health Initiative: A Longitudinal Study from the United States
Source: PLoS Med. 2016 Aug 16;13(8):e1002081. doi: 10.1371/journal.pmed.1002081 (PMC4987008; doi:10.1371/journal.pmed.1002081)
Supplement: S1 Appendix — (DOCX) [file pmed.1002081.s001.docx]

**Supplementary Appendix:**

**Duration of adulthood overweight, obesity, and cancer risk in the Women’s Health Initiative: potential for prevention**

Melina Arnold, Luohua Jiang, Marcia Stefanick, Karen C. Johnson, Dorothy S. Lane, Erin S. LeBlanc, Ross Prentice, Thomas Rohan, Beverly M. Snively, Mara Vitolins, Oleg Zaslavsky, Isabelle Soerjomataram, Hoda Anton-Culver

| **Table of Contents** |  |
| --- | --- |
| **Fig A.** Association between weighted cumulative overweight (BMI≥25) years (OWY) duration since age 18 and site-specific cancer risk, allowing for non-linear effects, with 95% CIs | 2 |
| **Fig B.** Crude incidence rates per 10,000 person-years for endometrial and postmenopausal breast cancer, by postmenopausal hormone use. | 3 |
| **Table A.** Hazard ratios (HR) of site-specific cancer related to overweight (BMI≥25) and obesity (BMI≥30) duration and intensity | 4 |
| **Table B.** Secondary analysis by postmenopausal hormone use | 6 |
| **Table C.** Secondary analysis by hysterectomy and oophorectomy status at recruitment | 7 |
| **Table D.** Secondary analysis by ethnicity | 8 |
| **Table E**. Secondary analysis by diabetes status | 9 |
| **Table F.** Secondary analysis by smoking status | 10 |
| **Table G.** Secondary analysis, using only self-reported BMI | 11 |
| **Short List of WHI Investigators** | 13 |

**Fig A.** Association between weighted cumulative overweight (BMI≥25) years (OWY) duration since age 18 and site-specific cancer risk, allowing for non-linear effects, with 95% CIs


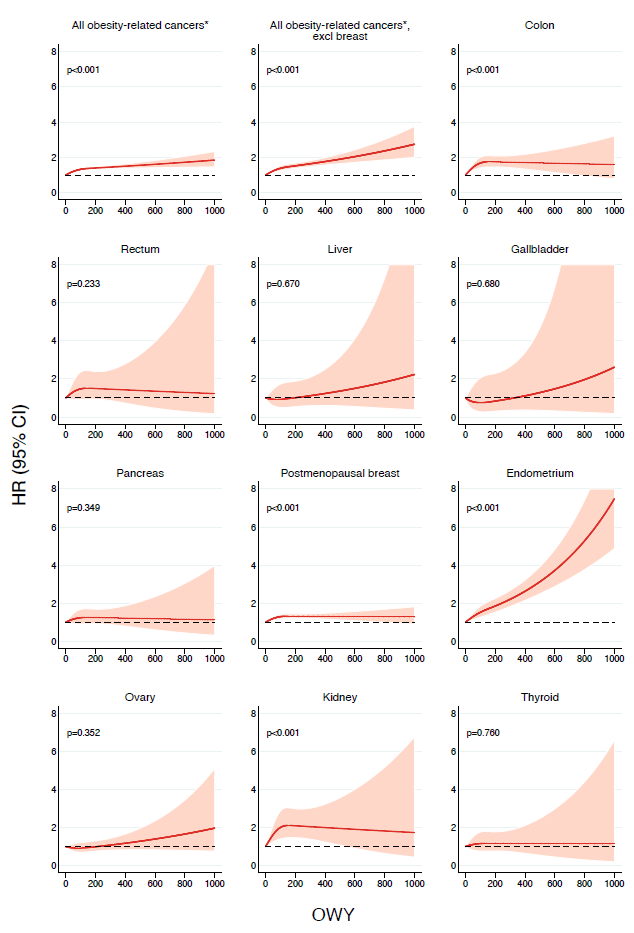


*All obesity-related sites comprise postmenopausal breast cancer as well as cancer of the colon, rectum, liver, gallbladder, pancreas, endometrium, ovary, kidney and thyroid

Hazard ratios (HR) are adjusted for age, ethnicity, education, physical activity, smoking status, dietary intake (in kcal) and diet quality score. Restricted cubic splines were fitted with knots at 0, 3 and 178 OWY. P-values are for non-linearity.

**Fig B.** Crude incidence rates per 10,000 person-years for endometrial and postmenopausal breast cancer, by postmenopausal hormone use.


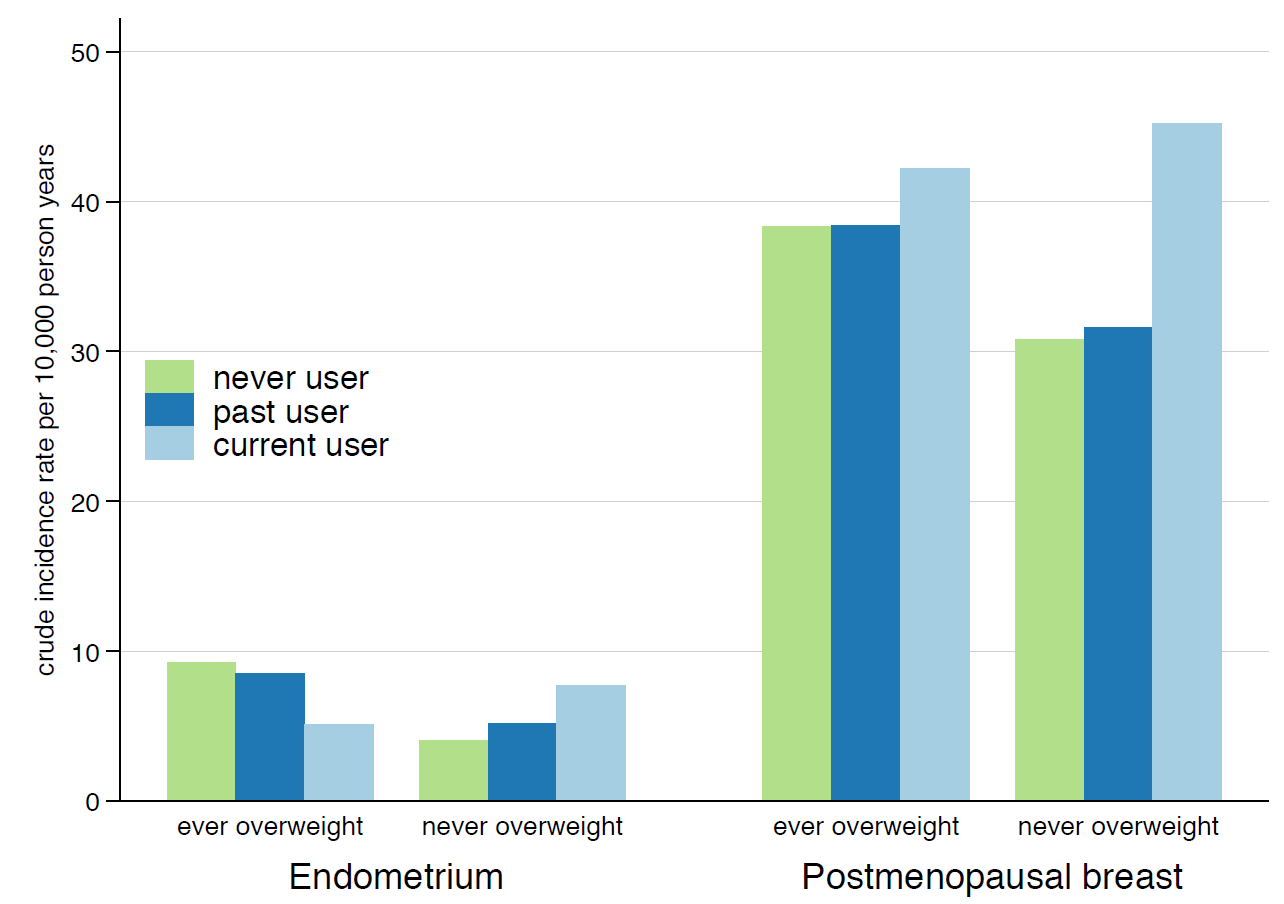


| **Table A.** Hazard ratios (HR) of site-specific cancer related to overweight (BMI≥25) and obesity (BMI≥30) duration and intensity | | | | |
| --- | --- | --- | --- | --- |
|  | -------- Model 1 ------- | -------- Model 2 ------- | -------- Model 3 ------- | -------- Model 4 ------- |
|  | HR 95% CI | HR 95% CI | HR 95% CI | HR 95% CI |
|  |  |  |  |  |
| **All obesity-related sites*** |  |  |  |  |
| Overweight duration, per 10yrs | 1.06 (1.05-1.08) | 1.07 (1.06-1.09) | 1.07 (1.06-1.09) |  |
| Obesity duration, per 10yrs | 1.11 (1.08-1.13) | 1.13 (1.10-1.16) | 1.12 (1.09-1.15) |  |
| OWY, per 100 units | 1.09 (1.07-1.11) | 1.10 (1.08-1.12) | 1.10 (1.08-1.12) |  |
| OBY, per 100 units | 1.11 (1.08-1.14) | 1.13 (1.09-1.16) | 1.12 (1.08-1.15) |  |
|  |  |  |  |  |
| **All obesity-related sites*, excl breast** | |  |  |  |
| Overweight duration, per 10yrs | 1.09 (1.06-1.11) | 1.10 (1.07-1.12) | 1.09 (1.07-1.12) |  |
| Obesity duration, per 10yrs | 1.17 (1.13-1.21) | 1.18 (1.14-1.22) | 1.17 (1.13-1.22) |  |
| OWY, per 100 units | 1.13 (1.10-1.16) | 1.14 (1.11-1.17) | 1.13 (1.10-1.16) |  |
| OBY, per 100 units | 1.17 (1.12-1.21) | 1.17 (1.13-1.22) | 1.17 (1.12-1.21) |  |
|  |  |  |  |  |
| **Colon** |  |  |  |  |
| Overweight duration, per 10yrs | 1.12 (1.08-1.16) | 1.12 (1.08-1.17) | 1.12 (1.08-1.17) | 1.11 (1.06-1.16) |
| Obesity duration, per 10yrs | 1.17 (1.10-1.24) | 1.17 (1.09-1.24) | 1.16 (1.09-1.24) | 1.14 (1.06-1.22) |
| OWY, per 100 units | 1.12 (1.07-1.18) | 1.12 (1.07-1.18) | 1.12 (1.06-1.17) | 1.10 (1.05-1.16) |
| OBY, per 100 units | 1.11 (1.02-1.21) | 1.10 (1.01-1.21) | 1.09 (1.00-1.20) | 1.07 (0.97-1.18) |
|  |  |  |  |  |
| **Rectum** |  |  |  |  |
| Overweight duration, per 10yrs | 1.08 (0.97-1.20) | 1.08 (0.97-1.20) | 1.09 (0.97-1.21) |  |
| Obesity duration, per 10yrs | 1.05 (0.87-1.27) | 1.05 (0.86-1.27) | 1.05 (0.86-1.28) |  |
| OWY, per 100 units | 1.08 (0.94-1.24) | 1.08 (0.93-1.24) | 1.08 (0.94-1.25) |  |
| OBY, per 100 units | 1.09 (0.86-1.39) | 1.08 (0.84-1.39) | 1.09 (0.85-1.40) |  |
|  |  |  |  |  |
| **Liver** |  |  |  |  |
| Overweight duration, per 10yrs | 1.00 (0.87-1.14) | 0.98 (0.85-1.12) | 0.96 (0.83-1.11) |  |
| Obesity duration, per 10yrs | 1.12 (0.90-1.39) | 1.09 (0.87-1.37) | 1.06 (0.84-1.33) |  |
| OWY, per 100 units | 1.11 (0.95-1.31) | 1.09 (0.92-1.29) | 1.07 (0.89-1.27) |  |
| OBY, per 100 units | 1.21 (0.99-1.48) | 1.19 (0.97-1.47) | 1.17 (0.93-1.46) |  |
|  |  |  |  |  |
| **Gallbladder** |  |  |  |  |
| Overweight duration, per 10yrs | 1.01 (0.81-1.26) | 0.98 (0.78-1.23) | 0.95 (0.75-1.20) |  |
| Obesity duration, per 10yrs | 1.25 (0.92-1.71) | 1.22 (0.88-1.67) | 1.18 (0.85-1.64) |  |
| OWY, per 100 units | 1.12 (0.87-1.45) | 1.09 (0.83-1.43) | 1.06 (0.79-1.43) |  |
| OBY, per 100 units | 1.15 (0.77-1.73) | 1.11 (0.73-1.71) | 1.09 (0.68-1.75) |  |
|  |  |  |  |  |
| **Pancreas** |  |  |  |  |
| Overweight duration, per 10yrs | 1.04 (0.97-1.11) | 1.04 (0.98-1.12) | 1.05 (0.98-1.12) | 1.05 (0.98-1.12) |
| Obesity duration, per 10yrs | 1.02 (0.91-1.15) | 1.03 (0.91-1.16) | 1.04 (0.91-1.17) | 1.03 (0.91-1.17) |
| OWY, per 100 units | 1.04 (0.94-1.14) | 1.04 (0.94-1.15) | 1.05 (0.95-1.16) | 1.05 (0.95-1.16) |
| OBY, per 100 units | 1.05 (0.89-1.25) | 1.06 (0.89-1.25) | 1.07 (0.90-1.26) | 1.06 (0.89-1.26) |
|  |  |  |  |  |
| **Postmenopausal breast** |  |  |  |  |
| Overweight duration, per 10yrs | 1.04 (1.02-1.06) | 1.06 (1.04-1.08) | 1.05 (1.03-1.07) | 1.06 (1.04-1.09) |
| Obesity duration, per 10yrs | 1.06 (1.03-1.10) | 1.09 (1.05-1.13) | 1.08 (1.05-1.12) | 1.11 (1.06-1.15) |
| OWY, per 100 units | 1.05 (1.03-1.08) | 1.07 (1.05-1.10) | 1.07 (1.04-1.10) | 1.09 (1.05-1.12) |
| OBY, per 100 units | 1.06 (1.01-1.11) | 1.08 (1.03-1.13) | 1.07 (1.02-1.12) | 1.11 (1.05-1.16) |
|  |  |  |  |  |
| **Endometrium** |  |  |  |  |
| Overweight duration, per 10yrs | 1.14 (1.09-1.19) | 1.17 (1.12-1.23) | 1.17 (1.12-1.22) | 1.16 (1.10-1.22) |
| Obesity duration, per 10yrs | 1.33 (1.25-1.41) | 1.38 (1.30-1.46) | 1.37 (1.29-1.46) | 1.37 (1.27-1.47) |
| OWY, per 100 units | 1.22 (1.17-1.26) | 1.24 (1.19-1.29) | 1.23 (1.18-1.28) | 1.21 (1.16-1.27) |
| OBY, per 100 units | 1.28 (1.21-1.35) | 1.30 (1.23-1.37) | 1.29 (1.22-1.36) | 1.25 (1.18-1.33) |
|  |  |  |  |  |
| **Ovary** |  |  |  |  |
| Overweight duration, per 10yrs | 0.98 (0.92-1.04) | 0.99 (0.93-1.06) | 0.99 (0.93-1.06) | 0.96 (0.89-1.04) |
| Obesity duration, per 10yrs | 0.99 (0.89-1.11) | 1.01 (0.90-1.13) | 1.02 (0.91-1.14) | 0.97 (0.84-1.13) |
| OWY, per 100 units | 1.02 (0.93-1.12) | 1.04 (0.95-1.14) | 1.05 (0.95-1.14) | 0.97 (0.85-1.11) |
| OBY, per 100 units | 1.09 (0.95-1.25) | 1.11 (0.97-1.27) | 1.12 (0.98-1.28) | 1.01 (0.81-1.26) |
|  |  |  |  |  |
| **Kidney** |  |  |  |  |
| Overweight duration, per 10yrs | 1.18 (1.09-1.28) | 1.17 (1.08-1.27) | 1.16 (1.07-1.26) |  |
| Obesity duration, per 10yrs | 1.20 (1.07-1.36) | 1.18 (1.04-1.34) | 1.17 (1.02-1.33) |  |
| OWY, per 100 units | 1.16 (1.07-1.27) | 1.15 (1.05-1.26) | 1.14 (1.04-1.25) |  |
| OBY, per 100 units | 1.16 (1.00-1.35) | 1.14 (0.97-1.34) | 1.13 (0.96-1.33) |  |
|  |  |  |  |  |
| **Thyroid** |  |  |  |  |
| Overweight duration, per 10yrs | 1.00 (0.91-1.10) | 1.02 (0.92-1.12) | 1.02 (0.92-1.13) |  |
| Obesity duration, per 10yrs | 1.05 (0.89-1.23) | 1.08 (0.92-1.27) | 1.08 (0.91-1.28) |  |
| OWY, per 100 units | 1.02 (0.88-1.17) | 1.04 (0.91-1.19) | 1.04 (0.90-1.20) |  |
| OBY, per 100 units | 1.00 (0.77-1.30) | 1.03 (0.79-1.33) | 1.02 (0.79-1.33) |  |
|  |  |  |  |  |
|  |  |  |  |  |
| Model 1: adjusted for age |  |  |  |  |
| Model 2: additionally adjusted for ethnicity and education | | |  |  |
| Model 3: additionally adjusted for smoking status, physical activity, energy intake and diet score | | | |  |
| Model 4: additionally adjusted for age at first birth, age at menopause, parity and hormone use (for breast, endometrial and ovarian cancer), red meat intake (colon cancer) and diabetes (pancreatic and colon cancer) | | | | |

| **Table B.** Secondary analysis by postmenopausal hormone use | | |  |  |
| --- | --- | --- | --- | --- |
|  | -------- Model 1 ------- | -------- Model 2 ------- | -------- Model 3 ------- | -------- Model 4 ------- |
|  | HR 95% CI | HR 95% CI | HR 95% CI | HR 95% CI |
|  |  |  |  |  |
| **Postmenopausal breast** |  |  |  |  |
| Overweight duration, per 10yrs |  |  |  |  |
| never user | 1.08 (1.04-1.12) | 1.10 (1.06-1.14) | 1.10 (1.06-1.14) | 1.11 (1.06-1.16) |
| past user | 1.07 (1.02-1.12) | 1.08 (1.03-1.13) | 1.07 (1.02-1.12) | 1.06 (1.00-1.12) |
| current user | 1.03 (1.00-1.06) | 1.04 (1.02-1.07) | 1.04 (1.01-1.07) | 1.04 (1.00-1.07) |
| Obesity duration, per 10yrs |  |  |  |  |
| never user | 1.11 (1.06-1.17) | 1.14 (1.08-1.20) | 1.14 (1.08-1.20) | 1.17 (1.10-1.24) |
| past user | 1.09 (1.01-1.17) | 1.10 (1.02-1.18) | 1.08 (1.00-1.16) | 1.08 (0.98-1.18) |
| current user | 1.05 (1.00-1.11) | 1.08 (1.02-1.13) | 1.06 (1.01-1.12) | 1.06 (1.00-1.13) |
| OWY, per 100 units |  |  |  |  |
| never user | 1.08 (1.04-1.12) | 1.10 (1.05-1.14) | 1.10 (1.05-1.14) | 1.12 (1.07-1.17) |
| past user | 1.08 (1.02-1.15) | 1.09 (1.03-1.16) | 1.08 (1.01-1.14) | 1.08 (1.01-1.16) |
| current user | 1.05 (1.00-1.09) | 1.07 (1.02-1.11) | 1.06 (1.01-1.10) | 1.06 (1.01-1.11) |
| OBY, per 100 units |  |  |  |  |
| never user | 1.07 (1.00-1.15) | 1.09 (1.01-1.17) | 1.09 (1.01-1.17) | 1.12 (1.04-1.20) |
| past user | 1.10 (0.99-1.22) | 1.11 (1.00-1.23) | 1.08 (0.97-1.20) | 1.11 (0.98-1.26) |
| current user | 1.07 (1.00-1.15) | 1.09 (1.02-1.17) | 1.08 (1.00-1.16) | 1.08 (0.99-1.17) |
|  |  |  |  |  |
| **Endometrium** |  |  |  |  |
| Overweight duration, per 10yrs |  |  |  |  |
| never user | 1.31 (1.21-1.42) | 1.35 (1.25-1.47) | 1.34 (1.24-1.46) | 1.30 (1.19-1.43) |
| past user | 1.24 (1.13-1.36) | 1.27 (1.15-1.40) | 1.30 (1.18-1.43) | 1.32 (1.18-1.48) |
| current user | 0.98 (0.91-1.05) | 1.00 (0.93-1.07) | 0.99 (0.92-1.07) | 1.00 (0.92-1.09) |
| Obesity duration, per 10yrs |  |  |  |  |
| never user | 1.45 (1.33-1.58) | 1.51 (1.38-1.66) | 1.50 (1.36-1.64) | 1.46 (1.32-1.63) |
| past user | 1.43 (1.28-1.61) | 1.48 (1.31-1.66) | 1.52 (1.35-1.71) | 1.52 (1.31-1.76) |
| current user | 1.07 (0.94-1.22) | 1.11 (0.97-1.26) | 1.10 (0.96-1.25) | 1.14 (0.98-1.33) |
| OWY, per 100 units |  |  |  |  |
| never user | 1.26 (1.19-1.32) | 1.28 (1.21-1.35) | 1.27 (1.20-1.34) | 1.27 (1.19-1.35) |
| past user | 1.30 (1.21-1.40) | 1.32 (1.22-1.42) | 1.32 (1.22-1.42) | 1.31 (1.19-1.44) |
| current user | 1.05 (0.94-1.17) | 1.08 (0.97-1.20) | 1.07 (0.96-1.19) | 1.09 (0.97-1.23) |
| OBY, per 100 units |  |  |  |  |
| never user | 1.30 (1.20-1.40) | 1.32 (1.22-1.42) | 1.31 (1.21-1.41) | 1.31 (1.20-1.43) |
| past user | 1.41 (1.27-1.57) | 1.42 (1.28-1.57) | 1.41 (1.27-1.56) | 1.37 (1.18-1.59) |
| current user | 1.12 (0.95-1.32) | 1.15 (0.99-1.34) | 1.14 (0.97-1.33) | 1.16 (0.99-1.36) |
|  |  |  |  |  |
| **Ovary** |  |  |  |  |
| Overweight duration, per 10yrs |  |  |  |  |
| never user | 0.97 (0.86-1.09) | 0.97 (0.86-1.10) | 0.97 (0.86-1.10) | 0.94 (0.81-1.10) |
| past user | 0.94 (0.82-1.09) | 0.96 (0.83-1.11) | 0.97 (0.84-1.12) | 0.95 (0.79-1.13) |
| current user | 1.01 (0.92-1.10) | 1.02 (0.93-1.11) | 1.03 (0.94-1.12) | 0.98 (0.88-1.09) |
| Obesity duration, per 10yrs |  |  |  |  |
| never user | 1.06 (0.88-1.26) | 1.07 (0.89-1.28) | 1.07 (0.89-1.29) | 1.01 (0.79-1.28) |
| past user | 0.87 (0.65-1.17) | 0.90 (0.67-1.21) | 0.91 (0.68-1.23) | 0.91 (0.64-1.30) |
| current user | 1.00 (0.84-1.19) | 1.02 (0.86-1.21) | 1.03 (0.86-1.23) | 0.95 (0.76-1.19) |
| OWY, per 100 units |  |  |  |  |
| never user | 1.10 (0.97-1.24) | 1.11 (0.98-1.26) | 1.11 (0.98-1.26) | 1.00 (0.81-1.23) |
| past user | 0.90 (0.70-1.16) | 0.93 (0.72-1.19) | 0.94 (0.73-1.21) | 0.94 (0.70-1.28) |
| current user | 1.00 (0.87-1.16) | 1.03 (0.89-1.19) | 1.03 (0.89-1.20) | 0.95 (0.78-1.16) |
| OBY, per 100 units |  |  |  |  |
| never user | 1.21 (1.04-1.40) | 1.22 (1.05-1.41) | 1.22 (1.05-1.41) | 1.05 (0.77-1.44) |
| past user | 0.82 (0.47-1.43) | 0.86 (0.50-1.49) | 0.88 (0.51-1.52) | 0.99 (0.59-1.68) |
| current user | 1.00 (0.75-1.33) | 1.03 (0.78-1.36) | 1.04 (0.78-1.37) | 0.94 (0.62-1.43) |
|  |  |  |  |  |
| Model 1: adjusted for age |  |  |  |  |
| Model 2: additionally adjusted for ethnicity and education | | |  |  |
| Model 3: additionally adjusted for smoking status, physical activity, energy intake and diet score | | | |  |
| Model 4: additionally adjusted for age at first birth, age at menopause and parity | | | |  |

| **Table C.** Secondary analysis by hysterectomy and oophorectomy status at recruitment | | | | |
| --- | --- | --- | --- | --- |
|  | -------- Model 1 ------- | -------- Model 2 ------- | -------- Model 3 ------- | -------- Model 4 ------- |
|  | HR 95% CI | HR 95% CI | HR 95% CI | HR 95% CI |
|  |  |  |  |  |
| **Postmenopausal breast** |  |  |  |  |
| Overweight duration, per 10yrs |  |  |  |  |
| never hysterectomy & never oophorectomy | 1.06 (1.03-1.08) | 1.07 (1.05-1.10) | 1.07 (1.04-1.10) | 1.08 (1.05-1.11) |
| never hysterectomy & ever oophorectomy | 1.02 (0.98-1.07) | 1.03 (0.99-1.08) | 1.02 (0.97-1.07) | 1.02 (0.96-1.08) |
| ever hysterectomy & ever oophorectomy | 1.02 (0.98-1.07) | 1.04 (0.99-1.09) | 1.03 (0.98-1.08) | 1.04 (0.99-1.10) |
| Obesity duration, per 10yrs |  |  |  |  |
| never hysterectomy & never oophorectomy | 1.08 (1.04-1.12) | 1.10 (1.06-1.15) | 1.10 (1.05-1.14) | 1.12 (1.07-1.18) |
| never hysterectomy & ever oophorectomy | 1.03 (0.96-1.12) | 1.05 (0.97-1.14) | 1.03 (0.95-1.11) | 1.05 (0.95-1.15) |
| ever hysterectomy & ever oophorectomy | 1.09 (1.01-1.17) | 1.11 (1.03-1.20) | 1.11 (1.02-1.20) | 1.12 (1.03-1.23) |
| OWY, per 100 units |  |  |  |  |
| never hysterectomy & never oophorectomy | 1.06 (1.03-1.10) | 1.08 (1.05-1.12) | 1.08 (1.04-1.11) | 1.10 (1.06-1.15) |
| never hysterectomy & ever oophorectomy | 1.03 (0.97-1.10) | 1.05 (0.98-1.11) | 1.03 (0.96-1.10) | 1.05 (0.97-1.13) |
| ever hysterectomy & ever oophorectomy | 1.07 (1.01-1.14) | 1.09 (1.02-1.16) | 1.09 (1.02-1.16) | 1.09 (1.02-1.17) |
| OBY, per 100 units |  |  |  |  |
| never hysterectomy & never oophorectomy | 1.06 (1.00-1.12) | 1.08 (1.02-1.14) | 1.07 (1.01-1.14) | 1.12 (1.05-1.19) |
| never hysterectomy & ever oophorectomy | 1.02 (0.91-1.15) | 1.04 (0.92-1.17) | 1.01 (0.90-1.15) | 1.06 (0.92-1.21) |
| ever hysterectomy & ever oophorectomy | 1.12 (1.02-1.24) | 1.14 (1.04-1.25) | 1.14 (1.03-1.26) | 1.13 (1.01-1.26) |
|  |  |  |  |  |
| **Endometrium (never hysterectomy & never oophorectomy)** | |  |  |  |
| Overweight duration, per 10yrs | 1.17 (1.12-1.23) | 1.19 (1.14-1.24) | 1.19 (1.13-1.24) | 1.19 (1.12-1.25) |
| Obesity duration, per 10yrs | 1.37 (1.29-1.45) | 1.39 (1.31-1.48) | 1.39 (1.30-1.47) | 1.41 (1.31-1.52) |
| OWY, per 100 units | 1.23 (1.19-1.28) | 1.24 (1.20-1.29) | 1.24 (1.19-1.29) | 1.24 (1.19-1.30) |
| OBY, per 100 units | 1.29 (1.23-1.37) | 1.30 (1.24-1.37) | 1.29 (1.23-1.37) | 1.30 (1.22-1.38) |
|  |  |  |  |  |
| **Ovary** |  |  |  |  |
| Overweight duration, per 10yrs |  |  |  |  |
| never hysterectomy & never oophorectomy | 0.99 (0.92-1.07) | 1.00 (0.93-1.08) | 1.00 (0.92-1.08) | 0.94 (0.85-1.04) |
| never hysterectomy & ever oophorectomy | 0.98 (0.88-1.10) | 0.99 (0.88-1.11) | 1.00 (0.89-1.13) | 1.00 (0.87-1.16) |
| Obesity duration, per 10yrs |  |  |  |  |
| never hysterectomy & never oophorectomy | 1.03 (0.90-1.18) | 1.05 (0.92-1.20) | 1.05 (0.91-1.20) | 0.98 (0.82-1.18) |
| never hysterectomy & ever oophorectomy | 0.93 (0.74-1.16) | 0.92 (0.73-1.16) | 0.94 (0.75-1.19) | 0.90 (0.66-1.23) |
| OWY, per 100 units |  |  |  |  |
| never hysterectomy & never oophorectomy | 1.07 (0.97-1.18) | 1.09 (0.99-1.20) | 1.09 (0.98-1.20) | 0.99 (0.85-1.16) |
| never hysterectomy & ever oophorectomy | 0.92 (0.75-1.13) | 0.91 (0.74-1.13) | 0.94 (0.76-1.16) | 0.91 (0.69-1.20) |
| OBY, per 100 units |  |  |  |  |
| never hysterectomy & never oophorectomy | 1.16 (1.01-1.33) | 1.18 (1.04-1.35) | 1.18 (1.03-1.34) | 1.07 (0.85-1.35) |
| never hysterectomy & ever oophorectomy | 0.84 (0.54-1.33) | 0.83 (0.52-1.33) | 0.87 (0.55-1.37) | 0.78 (0.39-1.54) |
|  |  |  |  |  |
| Model 1: adjusted for age |  |  |  |  |
| Model 2: additionally adjusted for ethnicity and education | |  |  |  |
| Model 3: additionally adjusted for smoking status, physical activity, energy intake and diet score | | | |  |
| Model 4: additionally adjusted for age at first birth, age at menopause, hormone use and parity | | | |  |

| **Table D.** Secondary analysis by ethnicity | |  |  |  |
| --- | --- | --- | --- | --- |
|  | -------- Model 1 ------- | -------- Model 2 ------- | -------- Model 3 ------- | -------- Model 4 ------- |
|  | HR 95% CI | HR 95% CI | HR 95% CI | HR 95% CI |
|  |  |  |  |  |
| **Postmenopausal breast** |  |  |  |  |
| Overweight duration, per 10yrs |  |  |  |  |
| Black/African American | 1.06 (0.98-1.16) | 1.07 (0.98-1.17) | 1.08 (0.99-1.18) | 1.06 (0.94-1.19) |
| Non-Hispanic White | 1.04 (1.02-1.06) | 1.05 (1.03-1.07) | 1.05 (1.03-1.07) | 1.06 (1.03-1.08) |
|  |  |  |  |  |
| Obesity duration, per 10yrs |  |  |  |  |
| Black/African American | 1.08 (0.97-1.19) | 1.09 (0.98-1.21) | 1.10 (0.99-1.22) | 1.08 (0.95-1.25) |
| Non-Hispanic White | 1.07 (1.03-1.11) | 1.08 (1.05-1.12) | 1.08 (1.04-1.11) | 1.10 (1.05-1.15) |
|  |  |  |  |  |
| OWY, per 100 units |  |  |  |  |
| Black/African American | 1.05 (0.97-1.14) | 1.05 (0.97-1.14) | 1.06 (0.98-1.16) | 1.06 (0.95-1.18) |
| Non-Hispanic White | 1.06 (1.03-1.09) | 1.07 (1.04-1.10) | 1.06 (1.03-1.10) | 1.08 (1.05-1.12) |
|  |  |  |  |  |
| OBY, per 100 units |  |  |  |  |
| Black/African American | 1.05 (0.92-1.19) | 1.05 (0.92-1.20) | 1.06 (0.93-1.21) | 1.07 (0.91-1.25) |
| Non-Hispanic White | 1.07 (1.01-1.12) | 1.08 (1.03-1.14) | 1.07 (1.01-1.13) | 1.10 (1.04-1.17) |
|  |  |  |  |  |
| Model 1: adjusted for age |  |  |  |  |
| Model 2: additionally adjusted for education | |  |  |  |
| Model 3: additionally adjusted for smoking status, physical activity, energy intake and diet score | | | |  |
| Model 4: additionally adjusted for age at first birth, age at menopause, hormone use and parity | | | |  |

| **Table E.** Secondary analysis by diabetes status | | |  |
| --- | --- | --- | --- |
|  | -------- Model 1 ------- | -------- Model 2 ------- | -------- Model 3 ------- |
|  | HR 95% CI | HR 95% CI | HR 95% CI |
|  |  |  |  |
| **Colon** |  |  |  |
| Overweight duration, per 10yrs |  |  |  |
| never diabetes | 1.10 (1.06-1.15) | 1.10 (1.06-1.15) | 1.10 (1.05-1.15) |
| ever diabetes | 1.24 (1.07-1.43) | 1.25 (1.07-1.45) | 1.26 (1.09-1.47) |
| Obesity duration, per 10yrs |  |  |  |
| never diabetes | 1.13 (1.05-1.21) | 1.13 (1.05-1.22) | 1.12 (1.04-1.20) |
| ever diabetes | 1.26 (1.08-1.47) | 1.27 (1.08-1.48) | 1.28 (1.09-1.50) |
| OWY, per 100 units |  |  |  |
| never diabetes | 1.10 (1.04-1.16) | 1.10 (1.04-1.16) | 1.09 (1.03-1.16) |
| ever diabetes | 1.15 (1.04-1.28) | 1.15 (1.04-1.28) | 1.15 (1.04-1.28) |
| OBY, per 100 units |  |  |  |
| never diabetes | 1.06 (0.95-1.19) | 1.06 (0.94-1.19) | 1.04 (0.92-1.18) |
| ever diabetes | 1.16 (1.00-1.35) | 1.16 (0.99-1.35) | 1.15 (0.99-1.34) |
|  |  |  |  |
| **Pancreas** |  |  |  |
| Overweight duration, per 10yrs |  |  |  |
| never diabetes | 1.05 (0.98-1.12) | 1.06 (0.99-1.13) | 1.06 (0.99-1.14) |
| ever diabetes | 0.94 (0.72-1.23) | 0.90 (0.69-1.19) | 0.92 (0.69-1.22) |
| Obesity duration, per 10yrs |  |  |  |
| never diabetes | 1.02 (0.90-1.16) | 1.04 (0.91-1.18) | 1.04 (0.91-1.19) |
| ever diabetes | 1.02 (0.71-1.48) | 0.97 (0.66-1.42) | 1.01 (0.69-1.48) |
| OWY, per 100 units |  |  |  |
| never diabetes | 1.04 (0.94-1.15) | 1.05 (0.95-1.17) | 1.06 (0.95-1.17) |
| ever diabetes | 1.00 (0.72-1.37) | 0.95 (0.68-1.33) | 0.99 (0.71-1.37) |
| OBY, per 100 units |  |  |  |
| never diabetes | 1.06 (0.88-1.27) | 1.07 (0.89-1.28) | 1.07 (0.90-1.28) |
| ever diabetes | 1.02 (0.63-1.65) | 0.97 (0.58-1.61) | 1.02 (0.64-1.61) |
|  |  |  |  |
| Model 1: adjusted for age |  |  |  |
| Model 2: additionally adjusted for ethnicity and education | | |  |
| Model 3: additionally adjusted for smoking status, physical activity, energy intake and diet score | | | |

| **Table F.** Secondary analysis by smoking status | | | |
| --- | --- | --- | --- |
|  | -------- Model 1 ------- | -------- Model 2 ------- | -------- Model 3 ------- |
|  | HR 95% CI | HR 95% CI | HR 95% CI |
|  |  |  |  |
| **Colon** |  |  |  |
| Overweight duration, per 10yrs |  |  |  |
| Never Smoked | 1.11 (1.05-1.17) | 1.11 (1.05-1.18) | 1.11 (1.05-1.17) |
| Past Smoker | 1.12 (1.06-1.19) | 1.13 (1.06-1.20) | 1.12 (1.06-1.19) |
| Current Smoker | 1.20 (1.04-1.38) | 1.18 (1.02-1.37) | 1.19 (1.02-1.38) |
| Obesity duration, per 10yrs |  |  |  |
| Never Smoked | 1.11 (1.01-1.23) | 1.11 (1.00-1.22) | 1.10 (0.99-1.21) |
| Past Smoker | 1.19 (1.09-1.31) | 1.20 (1.09-1.32) | 1.19 (1.08-1.31) |
| Current Smoker | 1.35 (1.12-1.63) | 1.35 (1.11-1.64) | 1.38 (1.12-1.69) |
| OWY, per 100 units |  |  |  |
| Never Smoked | 1.11 (1.03-1.19) | 1.10 (1.02-1.19) | 1.09 (1.01-1.18) |
| Past Smoker | 1.12 (1.04-1.20) | 1.13 (1.05-1.21) | 1.12 (1.04-1.21) |
| Current Smoker | 1.23 (1.07-1.42) | 1.23 (1.06-1.42) | 1.25 (1.07-1.44) |
| OBY, per 100 units |  |  |  |
| Never Smoked | 1.07 (0.93-1.24) | 1.06 (0.92-1.23) | 1.05 (0.91-1.23) |
| Past Smoker | 1.10 (0.97-1.26) | 1.11 (0.97-1.27) | 1.09 (0.95-1.26) |
| Current Smoker | 1.30 (1.05-1.61) | 1.29 (1.04-1.62) | 1.32 (1.05-1.64) |
|  |  |  |  |
| **Pancreas** |  |  |  |
| Overweight duration, per 10yrs |  |  |  |
| Never Smoked | 1.00 (0.91-1.10) | 1.00 (0.90-1.10) | 1.00 (0.91-1.11) |
| Past Smoker | 1.03 (0.93-1.14) | 1.05 (0.95-1.16) | 1.05 (0.95-1.16) |
| Current Smoker | 1.35 (1.08-1.69) | 1.35 (1.07-1.71) | 1.38 (1.09-1.75) |
| Obesity duration, per 10yrs |  |  |  |
| Never Smoked | 0.87 (0.70-1.09) | 0.86 (0.69-1.07) | 0.87 (0.69-1.08) |
| Past Smoker | 1.02 (0.85-1.22) | 1.05 (0.88-1.26) | 1.04 (0.87-1.26) |
| Current Smoker | 1.57 (1.23-2.02) | 1.60 (1.23-2.09) | 1.61 (1.23-2.11) |
| OWY, per 100 units |  |  |  |
| Never Smoked | 0.91 (0.75-1.09) | 0.89 (0.73-1.08) | 0.90 (0.74-1.09) |
| Past Smoker | 1.03 (0.89-1.19) | 1.06 (0.92-1.22) | 1.05 (0.91-1.22) |
| Current Smoker | 1.36 (1.17-1.58) | 1.39 (1.17-1.64) | 1.45 (1.21-1.75) |
| OBY, per 100 units |  |  |  |
| Never Smoked | 0.66 (0.36-1.18) | 0.63 (0.34-1.15) | 0.64 (0.35-1.18) |
| Past Smoker | 1.07 (0.85-1.35) | 1.11 (0.88-1.39) | 1.10 (0.87-1.38) |
| Current Smoker | 1.47 (1.19-1.81) | 1.51 (1.20-1.90) | 1.63 (1.26-2.12) |
|  |  |  |  |
| Model 1: adjusted for age |  |  |  |
| Model 2: additionally adjusted for ethnicity and education | | |  |
| Model 3: additionally adjusted for physical activity, energy intake and diet score | | | |

| **Table G.** Secondary analysis, using only self-reported BMI | | | |
| --- | --- | --- | --- |
|  | -------- Model 1 ------- | -------- Model 2 ------- | -------- Model 3 ------- |
|  | HR 95% CI | HR 95% CI | HR 95% CI |
|  |  |  |  |
| **All obesity-related sites*** |  |  |  |
| Overweight duration, per 10yrs | 1.05 (1.03-1.06) | 1.06 (1.05-1.08) | 1.06 (1.04-1.07) |
| Obesity duration, per 10yrs | 1.09 (1.06-1.12) | 1.11 (1.08-1.14) | 1.10 (1.07-1.13) |
| OWY, per 100 units | 1.06 (1.05-1.08) | 1.08 (1.06-1.10) | 1.07 (1.05-1.09) |
| OBY, per 100 units | 1.07 (1.04-1.10) | 1.08 (1.05-1.11) | 1.08 (1.05-1.11) |
|  |  |  |  |
| **All obesity-related sites*, excl breast** | |  |  |
| Overweight duration, per 10yrs | 1.07 (1.05-1.10) | 1.08 (1.06-1.11) | 1.08 (1.06-1.10) |
| Obesity duration, per 10yrs | 1.15 (1.11-1.19) | 1.16 (1.12-1.20) | 1.15 (1.11-1.19) |
| OWY, per 100 units | 1.10 (1.07-1.12) | 1.10 (1.08-1.13) | 1.10 (1.07-1.13) |
| OBY, per 100 units | 1.11 (1.07-1.15) | 1.11 (1.07-1.15) | 1.11 (1.07-1.15) |
|  |  |  |  |
| **Colon** |  |  |  |
| Overweight duration, per 10yrs | 1.11 (1.07-1.15) | 1.11 (1.07-1.15) | 1.11 (1.06-1.15) |
| Obesity duration, per 10yrs | 1.15 (1.08-1.23) | 1.15 (1.07-1.23) | 1.14 (1.06-1.22) |
| OWY, per 100 units | 1.09 (1.05-1.14) | 1.09 (1.04-1.14) | 1.09 (1.04-1.14) |
| OBY, per 100 units | 1.07 (0.98-1.16) | 1.06 (0.98-1.15) | 1.06 (0.97-1.15) |
|  |  |  |  |
| **Rectum** |  |  |  |
| Overweight duration, per 10yrs | 1.05 (0.95-1.17) | 1.05 (0.94-1.17) | 1.06 (0.95-1.18) |
| Obesity duration, per 10yrs | 1.00 (0.82-1.23) | 1.00 (0.81-1.23) | 1.00 (0.81-1.23) |
| OWY, per 100 units | 1.06 (0.92-1.21) | 1.05 (0.91-1.21) | 1.06 (0.91-1.22) |
| OBY, per 100 units | 1.06 (0.85-1.32) | 1.05 (0.84-1.33) | 1.06 (0.84-1.33) |
|  |  |  |  |
| **Liver** |  |  |  |
| Overweight duration, per 10yrs | 0.99 (0.86-1.13) | 0.96 (0.83-1.11) | 0.95 (0.82-1.09) |
| Obesity duration, per 10yrs | 1.11 (0.88-1.39) | 1.08 (0.86-1.36) | 1.04 (0.82-1.32) |
| OWY, per 100 units | 1.10 (0.95-1.27) | 1.08 (0.93-1.26) | 1.06 (0.90-1.25) |
| OBY, per 100 units | 1.15 (0.98-1.36) | 1.14 (0.96-1.35) | 1.13 (0.94-1.35) |
|  |  |  |  |
| **Gallbladder** |  |  |  |
| Overweight duration, per 10yrs | 1.03 (0.83-1.28) | 1.00 (0.80-1.25) | 0.97 (0.77-1.22) |
| Obesity duration, per 10yrs | 1.25 (0.92-1.70) | 1.21 (0.88-1.67) | 1.18 (0.85-1.64) |
| OWY, per 100 units | 1.09 (0.86-1.39) | 1.07 (0.82-1.38) | 1.04 (0.78-1.40) |
| OBY, per 100 units | 1.08 (0.71-1.63) | 1.04 (0.66-1.65) | 1.03 (0.62-1.71) |
|  |  |  |  |
| **Pancreas** |  |  |  |
| Overweight duration, per 10yrs | 1.01 (0.95-1.08) | 1.01 (0.95-1.09) | 1.02 (0.95-1.09) |
| Obesity duration, per 10yrs | 1.01 (0.89-1.15) | 1.02 (0.89-1.16) | 1.02 (0.90-1.16) |
| OWY, per 100 units | 1.01 (0.92-1.12) | 1.02 (0.92-1.13) | 1.02 (0.93-1.13) |
| OBY, per 100 units | 1.03 (0.88-1.21) | 1.03 (0.88-1.21) | 1.04 (0.89-1.21) |
|  |  |  |  |
| **Postmenopausal breast** |  |  |  |
| Overweight duration, per 10yrs | 1.03 (1.01-1.05) | 1.04 (1.02-1.06) | 1.04 (1.02-1.06) |
| Obesity duration, per 10yrs | 1.05 (1.01-1.08) | 1.07 (1.03-1.11) | 1.06 (1.02-1.10) |
| OWY, per 100 units | 1.04 (1.01-1.06) | 1.05 (1.03-1.08) | 1.05 (1.02-1.07) |
| OBY, per 100 units | 1.04 (0.99-1.08) | 1.05 (1.01-1.10) | 1.05 (1.00-1.09) |
|  |  |  |  |
| **Endometrium** |  |  |  |
| Overweight duration, per 10yrs | 1.13 (1.08-1.18) | 1.16 (1.11-1.21) | 1.15 (1.10-1.21) |
| Obesity duration, per 10yrs | 1.31 (1.23-1.39) | 1.36 (1.28-1.44) | 1.35 (1.27-1.43) |
| OWY, per 100 units | 1.17 (1.13-1.21) | 1.18 (1.14-1.22) | 1.17 (1.14-1.21) |
| OBY, per 100 units | 1.19 (1.13-1.24) | 1.20 (1.15-1.25) | 1.19 (1.14-1.25) |
|  |  |  |  |
| **Ovary** |  |  |  |
| Overweight duration, per 10yrs | 0.96 (0.90-1.02) | 0.97 (0.91-1.03) | 0.97 (0.91-1.04) |
| Obesity duration, per 10yrs | 0.96 (0.85-1.08) | 0.97 (0.86-1.10) | 0.98 (0.87-1.11) |
| OWY, per 100 units | 0.99 (0.91-1.09) | 1.01 (0.92-1.11) | 1.02 (0.92-1.12) |
| OBY, per 100 units | 1.05 (0.93-1.20) | 1.07 (0.95-1.21) | 1.08 (0.95-1.21) |
|  |  |  |  |
| **Kidney** |  |  |  |
| Overweight duration, per 10yrs | 1.18 (1.09-1.27) | 1.16 (1.07-1.26) | 1.16 (1.07-1.25) |
| Obesity duration, per 10yrs | 1.16 (1.02-1.33) | 1.14 (0.99-1.30) | 1.12 (0.98-1.29) |
| OWY, per 100 units | 1.12 (1.03-1.21) | 1.11 (1.01-1.21) | 1.10 (1.01-1.20) |
| OBY, per 100 units | 1.09 (0.94-1.26) | 1.07 (0.91-1.26) | 1.06 (0.90-1.26) |
|  |  |  |  |
| **Thyroid** |  |  |  |
| Overweight duration, per 10yrs | 1.00 (0.91-1.10) | 1.02 (0.92-1.12) | 1.02 (0.92-1.13) |
| Obesity duration, per 10yrs | 1.03 (0.87-1.22) | 1.06 (0.89-1.25) | 1.05 (0.88-1.26) |
| OWY, per 100 units | 1.00 (0.87-1.15) | 1.02 (0.89-1.18) | 1.02 (0.89-1.18) |
| OBY, per 100 units | 0.97 (0.74-1.27) | 1.00 (0.77-1.29) | 0.99 (0.76-1.30) |
|  |  |  |  |
| Model 1: adjusted for age |  |  |  |
| Model 2: additionally adjusted for ethnicity and education | | |  |
| Model 3: additionally adjusted for smoking status, physical activity, energy intake and diet score | | | |
| Model 4: additionally adjusted for age at first birth, age at menopause, parity and hormone use (for breast, endometrial and ovarian cancer), red meat intake (colon cancer) and diabetes (pancreatic and colon cancer) | | | |

**Short List of WHI Investigators**

**Program Office:** (National Heart, Lung, and Blood Institute, Bethesda, Maryland) Jacques Rossouw, Shari Ludlam, Dale Burwen, Joan McGowan, Leslie Ford, and Nancy Geller

**Clinical Coordinating Center:** Clinical Coordinating Center: (Fred Hutchinson Cancer Research Center, Seattle, WA) Garnet Anderson, Ross Prentice, Andrea LaCroix, and Charles Kooperberg

**Investigators and Academic Centers:** (Brigham and Women's Hospital, Harvard Medical School, Boston, MA) JoAnn E. Manson; (MedStar Health Research Institute/Howard University, Washington, DC) Barbara V. Howard; (Stanford Prevention Research Center, Stanford, CA) Marcia L. Stefanick; (The Ohio State University, Columbus, OH) Rebecca Jackson; (University of Arizona, Tucson/Phoenix, AZ) Cynthia A. Thomson; (University at Buffalo, Buffalo, NY) Jean Wactawski-Wende; (University of Florida, Gainesville/Jacksonville, FL) Marian Limacher; (University of Iowa, Iowa City/Davenport, IA) Robert Wallace; (University of Pittsburgh, Pittsburgh, PA) Lewis Kuller; (Wake Forest University School of Medicine, Winston-Salem, NC) Sally Shumaker

**Women’s Health Initiative Memory Study:** (Wake Forest University School of Medicine, Winston-Salem, NC) Sally Shumaker

For a list of all the investigators who have contributed to WHI science, please visit: https://www.whi.org/researchers/Documents%20%20Write%20a%20Paper/WHI%20Investigato r%20Long%20List.pdf
