# Supplementary material for: Duration of Adulthood Overweight, Obesity, and Cancer Risk in the Women’s Health Initiative: A Longitudinal Study from the United States
Source: PLoS Med. 2016 Aug 16;13(8):e1002081. doi: 10.1371/journal.pmed.1002081 (PMC4987008; doi:10.1371/journal.pmed.1002081)
Supplement: S1 Analysis Plan — (DOCX) [file pmed.1002081.s002.docx]

**Statistical analysis plan (excerpted from study proposal):**

**Duration of adulthood overweight, obesity, and cancer risk in the Women’s Health Initiative: potential for prevention**

Melina Arnold, Luohua Jiang, Marcia Stefanick, Karen C. Johnson, Dorothy S. Lane, Erin S. LeBlanc, Ross Prentice, Thomas Rohan, Beverly M. Snively, Mara Vitolins, Oleg Zaslavsky, Isabelle Soerjomataram, Hoda Anton-Culver

**OBJECTIVES**

The purpose of this study is to:

1. Estimate body mass index (BMI) growth curves across life course using repeated measurements of weight and height, applying a hierarchical linear model while adjusting for various baseline characteristics
2. Compute the years of life spent with obesity (LYO) and determine the influence of physical activity, diet (mean energy intake), smoking, alcohol, reproductive risk factors, socioeconomic status and ethnicity on LYO
3. Assess the relationship between LYO and the risk of developing (site-specific) cancer
4. Validate the relationship between BMI and cancer, taking into account the effects of study arm (CT vs. OS), different obesity measures (prospective, retrospective) and missing baseline characteristics.

**METHODS**

**Study population**

The Women’s Health Initiative (WHI) is a large, multi-center prospective cohort study of postmenopausal women. The WHI was designed to have a clinical trial (CT) arm and an observational study (OS) arm [1], which recruited postmenopausal women ages 50-79 to be followed for the development of the most common causes of death including cardiovascular disease and cancer. Details of the design of the study, as well as the baseline measures and reliability, have been published elsewhere [1-4]. In total, 93,676 women enrolled in the OS arm of the study and 68,132 enrolled in the CT arm (n=161,808) [4].

**Variable definition**

Our main dependent variables of interest are the anthropometric measurements ascertained using Forms 42, 80 and 143-148. Additional dependent variables are listed at the end of this document and include demographic characteristics, lifestyle factors (smoking/alcohol, physical activity, diet), reproductive history, general health indicators, diabetes, co-morbidities, medications, and family history of all primary outcomes of interest.

Diet intake will be approximated using total energy intake (kcal) per day and energy from (saturated) fat, derived from a semi-quantitative food-frequency questionnaire [5]. For colorectal cancer risk, daily servings of red meat and sodium intake (mg) will be used to account for confounding. Physical activity will be expressed based on the frequency and duration of several types of recreational activity and MET (metabolic equivalents) scores will be computed as the product of days per week, minutes per day, and the MET value for each activity [6]. Socioeconomic status will be estimated based on highest education, occupation and total family income.

Clinical outcomes (including cancer diagnoses) were updated annually in the OS and semi-annually in the CT by mailed or telephone questionnaires, and self-reported diagnoses were verified locally by WHI physician adjudicators [7]. Clinical outcomes of interest in this analysis include obesity-related cancers (breast, colorectum, ovary, endometrium, kidney, pancreas).

**Statistical analyses**

The analysis will be performed in four steps (corresponding to objectives 1-4):

1. Using all available BMI information from subjects with at least two BMI measurements (retrospective (Form 42): self-reported BMI at age 18, 35 and 50; prospective: at study baseline (measured, Form 80) and at follow-up (self-reported, Forms 143-148)) BMI will be modelled across ages for every cohort member using a quadratic growth model with a random intercept and random slope [8]. The model will be adjusted for important covariates that are associated with BMI, such as age, physical activity and smoking status. This method will allow individuals to have different intercepts and slopes, i.e. their own ‘BMI trajectory’. A more detailed description of the model construction is presented in Annex 1.
2. The predicted BMI during life course (across ages) from the model in step 1 will enable us to estimate the sum of years of life spent with overweight/obesity (LYO) per individual cohort member. We will thereby follow the methodology as applied by Stolzenberg-Solomon and colleagues in a recent study on lifetime adiposity and the risk of pancreatic cancer in the NIH-AARP Diet and Health Study cohort [9]. For cancer patients, the BMI information from the last year before study exit will be censored.
3. Finally, the relation between LYO and the risk to develop (site-specific) cancer will be assessed using cox proportional hazards regression with age as time metric. Smoothing of the data will be performed using cubic splines for age. Different models will be fitted to take into account confounders in a stepwise manner. This will include the following variables: ethnic group, socioeconomic status, physical activity, smoking, alcohol use, family history of cancer, (history of) blood pressure, (history of) diabetes, hormone replacement therapy (HRT) use and other reproductive risk factors. Subjects will be censored at study exit (death, lost to follow-up, any cancer diagnosis, end of study), whichever occurred first. Effect modification by smoking status, HRT use and diabetes will be also be tested by performing stratified analyses.
4. Sensitivity analyses will be carried out taking into account the effects of study arm (CT vs. OS), different obesity measures (prospective, retrospective) and missing baseline characteristics to test the robustness of our findings.

**Annex 1: Using hierarchical linear models to estimate growth curves for BMI**

**Model construction**

**First level (measurement occasion):**

$$Y_{\mathrm{ij}}= \beta_{0j}+ \beta_{1j}T_{\mathrm{ij}}+\beta_{2j}T_{\mathrm{ij}}^{2}+e_{\mathrm{ij}}$$

where

i individual

j measurement occasion

$Y_{\mathrm{ij}}$ predicted BMI for individual i at measurement occasion j

$T_{\mathrm{ij}}$ Length of time interval in years between baseline and the consecutive ith (i =1,2,…,nj) follow-up and for the jth (j=1,2,…,N) individual in the data set (fixed effect)

(i=1 for the baseline, meaning that T1j=0 for all j)

$e_{\mathrm{ij}}$ first-level residual (variance homogeneous, independent among subjects and different time points within subjects)

**Second level (individual):**

The random coefficients$\beta$’s are further modelled for the second level individuals by

decomposing into fixed and random components as follows (coefficients as outcome

analysis):

$$\beta_{0j}=\gamma_{00}+\gamma_{01}\mathrm{AGE}_{j}+\gamma_{02}\mathrm{SEX}_{j}+\gamma_{03}\mathrm{SMOKING}_{j}+\gamma_{04}{OTHER COVARIATES}_{j}+u_{0j}$$

$$\beta_{1j}=\gamma_{10}+\gamma_{11}\mathrm{AGE}_{j}+\gamma_{12}\mathrm{SEX}_{j}+\gamma_{13}\mathrm{SMOKING}_{j}+\gamma_{14}{OTHER COVARIATES}_{j}+u_{1j}$$

$$\beta_{2j}=\gamma_{20}+\gamma_{21}\mathrm{AGE}_{j}+\gamma_{22}\mathrm{SEX}_{j}+\gamma_{23}\mathrm{SMOKING}_{j}+\gamma_{24}{OTHER COVARIATES}_{j}+u_{2j}$$

where $u_{\mathrm{oj}}$, $u_{1j}$ and $u_{2j}$ represent residuals for intercepts, slope and quadratic terms, respectively (individual-level random effects).

**References**

1. Design of the Women's Health Initiative clinical trial and observational study. The Women's Health Initiative Study Group. Controlled clinical trials. 1998;19(1):61-109. PubMed PMID: 9492970.

2. Hays J, Hunt JR, Hubbell FA, Anderson GL, Limacher M, Allen C, et al. The Women's Health Initiative recruitment methods and results. Annals of epidemiology. 2003;13(9 Suppl):S18-77. PubMed PMID: 14575939.

3. Langer RD, White E, Lewis CE, Kotchen JM, Hendrix SL, Trevisan M. The Women's Health Initiative Observational Study: baseline characteristics of participants and reliability of baseline measures. Annals of epidemiology. 2003;13(9 Suppl):S107-21. PubMed PMID: 14575943.

4. Phipps AI, Chlebowski RT, Prentice R, McTiernan A, Stefanick ML, Wactawski-Wende J, et al. Body size, physical activity, and risk of triple-negative and estrogen receptor-positive breast cancer. Cancer Epidemiol Biomarkers Prev. 2011;20(3):454-63. doi: 10.1158/1055-9965.EPI-10-0974. PubMed PMID: 21364029; PubMed Central PMCID: PMC3064558.

5. Patterson RE, Kristal AR, Tinker LF, Carter RA, Bolton MP, Agurs-Collins T. Measurement characteristics of the Women's Health Initiative food frequency questionnaire. Ann Epidemiol. 1999;9(3):178-87. PubMed PMID: 10192650.

6. Ainsworth BE, Haskell WL, Herrmann SD, Meckes N, Bassett DR, Jr., Tudor-Locke C, et al. 2011 Compendium of Physical Activities: a second update of codes and MET values. Medicine and science in sports and exercise. 2011;43(8):1575-81. doi: 10.1249/MSS.0b013e31821ece12. PubMed PMID: 21681120.

7. Curb JD, McTiernan A, Heckbert SR, Kooperberg C, Stanford J, Nevitt M, et al. Outcomes ascertainment and adjudication methods in the Women's Health Initiative. Ann Epidemiol. 2003;13(9 Suppl):S122-8. PubMed PMID: 14575944.

8. Heo M, Faith MS, Mott JW, Gorman BS, Redden DT, Allison DB. Hierarchical linear models for the development of growth curves: an example with body mass index in overweight/obese adults. Stat Med. 2003;22(11):1911-42. doi: 10.1002/sim.1218. PubMed PMID: 12754724.

9. Stolzenberg-Solomon RZ, Schairer C, Moore S, Hollenbeck A, Silverman DT. Lifetime adiposity and risk of pancreatic cancer in the NIH-AARP Diet and Health Study cohort. Am J Clin Nutr. 2013;98(4):1057-65. doi: 10.3945/ajcn.113.058123. PubMed PMID: 23985810; PubMed Central PMCID: PMC3778860.
